# Spatiotemporal interaction of immune and renal cells controls glomerular crescent formation in autoimmune kidney disease

**DOI:** 10.1038/s41590-025-02291-8

**Published:** 2025-09-30

**Authors:** Zeba Sultana, Robin Khatri, Behnam Yousefi, Nikhat Shaikh, Saskia L. Jauch-Speer, Darius P. Schaub, Jonas Engesser, Malte Hellmig, Vincent Piegsa, Arthur Hube, Varshi Sivayoganathan, Alina Borchers, Anett Peters, Anna Kaffke, Stephanie Zielinski, Hans-Joachim Paust, Thiago Goldbeck-Strieder, Ulrich O. Wenzel, Victor G. Puelles, Elion Hoxha, Thorsten Wiech, Catherine Meyer-Schwesinger, Tobias B. Huber, Ulf Panzer, Stefan Bonn, Christian F. Krebs

**Affiliations:** 1 III. Department of Medicine, University Medical Center Hamburg-Eppendorf, Hamburg, Germany; 2 Hamburg Center for Translational Immunology (HCTI), University Medical Center Hamburg-Eppendorf, Hamburg, Germany; 3 Hamburg Center for Kidney Health (HCKH), University Medical Center Hamburg-Eppendorf, Hamburg, Germany; 4 Institute of Medical Systems Bioinformatics, Center for Molecular Neurobiology (ZMNH), University Medical Center Hamburg-Eppendorf, Hamburg, Germany; 5 Center for Biomedical AI (bAIome), University Medical Center Hamburg-Eppendorf, Hamburg, Germany; 6 German Center for Child and Adolescent Health (DZKJ), partner site Hamburg, University Medical Center Hamburg-Eppendorf, Hamburg, Germany; 7 Single Cell Core Facility, University Medical Center Hamburg-Eppendorf, Hamburg, Germany; 8 Institute of Cellular and Integrative Physiology, University Medical Center Hamburg-Eppendorf, Hamburg, Germany; 9 Institute of Pathology, Division of Nephropathology, University Medical Center Hamburg-Eppendorf, Hamburg, Germany

**Keywords:** Immunopathogenesis, Lupus nephritis, Autoimmune diseases

## Abstract

Rapidly progressive glomerulonephritis (RPGN) is the most aggressive group of autoimmune kidney diseases and is characterized by glomerular crescent formation with proliferation of parietal epithelial cells (PECs). However, the underlying mechanisms of glomerular crescent formation are incompletely understood. Here we provide a high-resolution spatial kidney cell atlas of 57 samples from patients with RPGN (ANCA-associated GN, lupus nephritis and anti-glomerular basement membrane-GN) to characterize the cell signaling pathways in glomerular crescent development. Early platelet-derived growth factor (PDGF) signaling from epithelial and mesangial cells caused PEC activation and proliferation in glomerular crescents, whereas later transforming growth factor (TGF)-β signaling from macrophages, T cells and epithelial and mesangial cells triggered expression of extracellular matrix components in PECs associated with glomerulosclerosis and disease progression. These findings were similar across the different GNs and were functionally validated in experimental GN by PDGF and TGFβ blockade. These results highlight a spatiotemporally conserved progression program into glomerular crescents and sclerosis and indicate new treatment options for autoimmune kidney disease.

## Abstract (web summary)

Here the authors provide a deep tissue analysis of patients with autoimmune kidney diseases, identifying a conserved spatiotemporal immune program for the development of glomerular crescents and sclerosis.

## Main

Rapidly progressive glomerulonephritis (RPGN) is a clinical syndrome characterized by a fast decline in kidney function accompanied by distinct histological features such as necrotizing glomerulonephritis and glomerular crescent formation. As a disease category, glomerulonephritis is among the most common causes for end-stage renal disease associated with a high morbidity and mortality. RPGN can develop in patients with systemic lupus erythematosus (SLE), ANCA-associated vasculitis (ANCA-GN) and anti-glomerular basement membrane antibody disease (anti-GBM). Infiltrating immune cells and parietal epithelial cells (PECs) of the glomerulus are considered to be major cellular contributors to glomerular crescents^1–7^. Although PECs usually build the Bowman’s capsule, they proliferate in crescentic glomerulonephritis (cGN) and contribute to the glomerular crescent. Signaling between immune cells and renal cells has been proposed as crucial in the immunopathogenesis of glomerulonephritis; however, a comprehensive definition of infiltrating immune cells, their precise localization and the cellular signaling that results in PEC proliferation and glomerulosclerosis remain missing.

To facilitate the spatial understanding of cellular and molecular disease processes, recent technical developments allow for the spatially resolved, multiplex transcriptional analysis of tissues^8^. These available technologies such as MERSCOPE, CosMx and Xenium measure messenger RNA using slightly different approaches, including signal amplification processes^9,10^. The Xenium platform relies on predefined oligonucleotide probes that are circularized and amplified after binding to the matching mRNA, resulting in an augmented detection signal. This enables the measurement of gene transcription at high resolution, which is important for the precise identification and spatial localization of specific cell types.

Here we used high-resolution spatial transcriptomics combined with multiplex spatial protein detection to investigate the development of glomerular crescents in the human kidney. We defined the glomerular niche, uncovered the cellular composition of the glomerulus plus the adjacent area and showed PECs to be major mechanistic hubs in RPGN etiology. A comparative analysis of cell interactions in cGN versus control conditions revealed strong signaling from PECs to other cells. The analysis of signaling pathways targeting PECs identified upregulated PDGF signaling originating from various glomerular cells and transforming growth factor (TGF)-β signaling from fibrotic mesangial and immune cells. In combination with crescent formation trajectory analysis, this defined the sequential upregulation of, first, platelet-derived growth factor (PDGF) and, later, TGFβ and the respective downstream signaling in PECs, resulting in cell proliferation and crescent formation, respectively. These findings could be confirmed in experimental cGN by blockade of PDGF with nintedanib and TGFβ with fostamatinib. The identification of PDGF and TGFβ in the sequence of crescent formation might provide a basis for targeted therapies in glomerulonephritis.

## Results

### Patient selection and study design

**Fig. 1:**
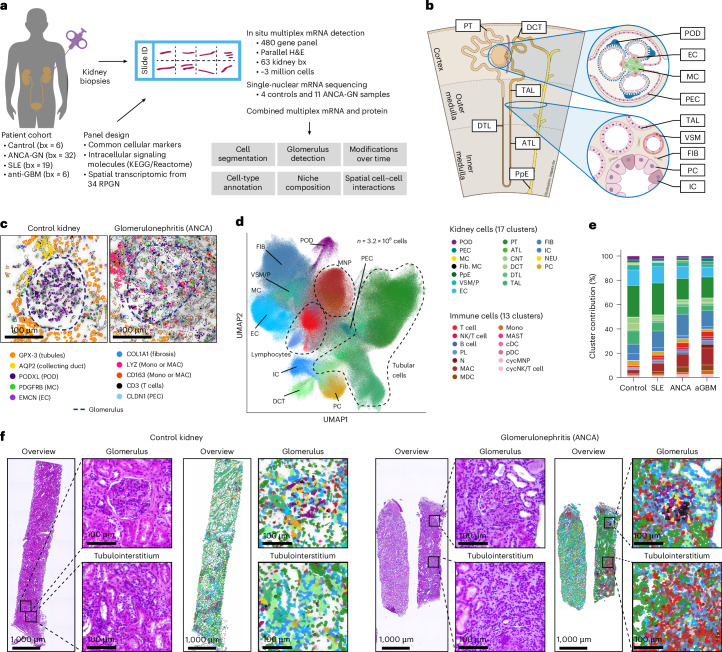
Major cell types in the human renal cortex in health and inflammation. **a**, Overview of the patient cohort from the Hamburg Glomerulonephritis Registry and the analysis performed. **b**, Schematic showing the cross-section of a human kidney tissue with the localization of various cell types in different regions. Inset: cross-section of a glomerulus (top) and tubulointerstitial region (bottom) and the cell types therein. **c**, DAPI-stained images from kidney biopsies of control (left) and a patient with ANCA-GN (right). The images show glomeruli and surrounding regions overlaid with cell boundaries determined using a cell segmentation algorithm. Localization of specific marker genes is indicated, with glomerular boundaries highlighted for clarity. **d**, Uniform Manifold Approximation and Projection (UMAP) of ~3.2 million cells retained after QC filtering. Cells are colored based on their annotated cell type. **e**, Stacked bar plot showing the proportions of different cell types from the complete biopsy tissues across the four disease conditions. **f**, images from control (left four images) and ANCA-GN (right four images) kidney biopsies showing a glomerulus and tubulointerstitial region. For each region, the adjoining plots show the segmented cells color coded according to their cell-type annotation, illustrating cellular composition differences between conditions in different regions of the tissue. aGBM, anti-GBM antibodies; ATL, ascending thin limb of loop of Henle; bx, biopsies; cDC, conventional dendritic cell; CNT, connecting tubule; cycNKC/T, cycling NK cytotoxic T cell; DCT, distal convoluted tubule; DTL, descending thin limb of loop of Henle; FIB, fibroblast; Fib. MC, fibrotic mesangial cell; IC, intercalated cell of collecting duct; KEGG, Kyoto Encyclopedia of Genes and Genomes; MAC, macrophage; MC, mesangial cell; MDC, monocyte-derived cell; Mono, monocyte; N, neutrophil; NEU, neuronal cell; PpE, papillary tip epithelial cells abutting the calyx; pDC, plasmacytoid dendritic cell; PL, plasma cell; POD, podocyte; PT, proximal tubule; TAL, thick ascending limb of loop of Henle; VSM/P, vascular smooth muscle cells or pericytes.

**Extended Data Fig. 1:**
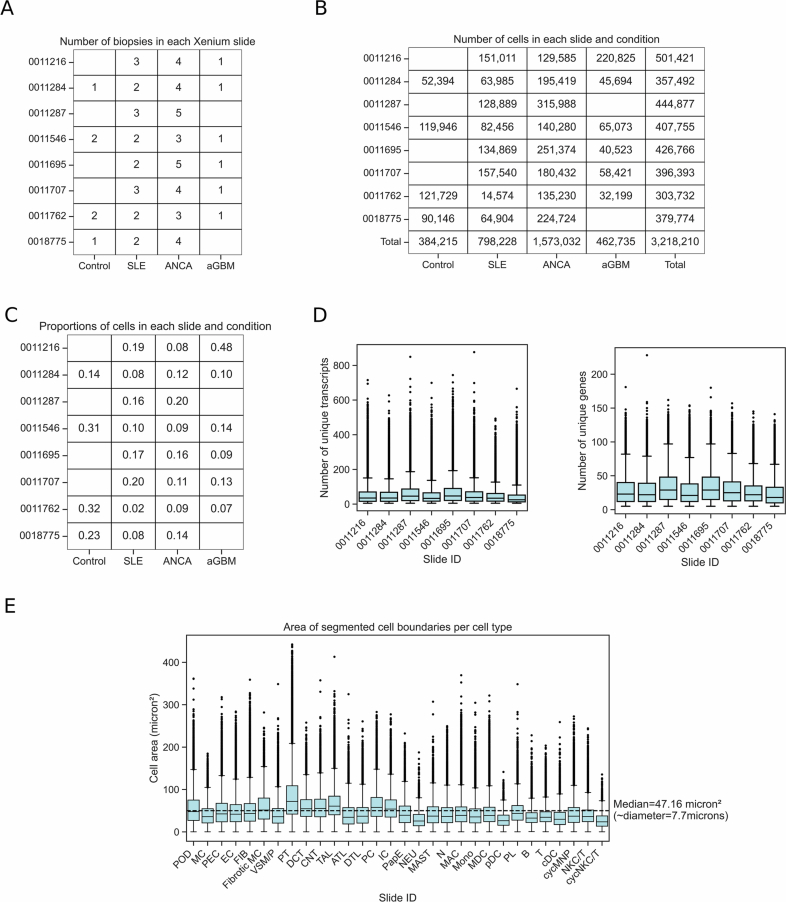
Quality control metrics and cellular characteristics of Xenium spatial transcriptomics data. **a**, Number of samples belonging to control and disease conditions across the 8 Xenium slides. **b-c**, Number (**b**) and distribution (**c**) of cells in each slide and condition after filtering for quality control. **d**, Boxplot showing distribution of number of unique transcripts (left) and number of unique genes (right) detected in each cell. The number of data points (cells per slide) used to create each boxplot are—Slide 0011216: 501,421, Slide 0011284: 357,492, Slide 0011287: 444,877, Slide 0011546: 407,755, Slide 0011695: 426,766, Slide 0011707: 396,393, Slide 0011762: 303,762, 0018775: 379,774. Boxplots show the median (middle horizontal line), interquartile range (box), Tukey-style whiskers (lines beyond the box), outliers (data points beyond 1.5*interquartile or below −1.5*interquartile). **e**, Boxplot showing distribution of segmented cell-areas per cell type. The median cell area and approximate diameter overall cells are indicated by a dashed line. The number of datapoints (cells per cell type) used to create boxplots are—POD: 61,319, MC: 26,349, PEC: 68,820, EC: 348,554, FIB: 478,776, Fibrotic MC: 10,288, VSM/P: 109,000, PT: 658,356, DCT: 57,590, CNT: 84,777, TAL: 222,773, ATL: 19,383, DTL: 51,333, PC: 100,663, IC: 92,864, PapE: 18,331, NEU: 5,028, MAST: 12,658, N: 41,570, MAC: 285,800, Mono: 55,537, MDC: 112,695, pDC: 4,164, PL: 61,013, B: 32,780, T: 106,672, cDC: 13,761, cycMNP: 30,547, NKC/T: 42,679, cycNKC/T: 4,130. Boxplots show the median (middle horizontal line), interquartile range (box), Tukey-style whiskers (lines beyond the box), outliers (data points beyond 1.5*interquartile or below −1.5*interquartile).

To understand the molecular pathogenesis of RPGN, we conducted a comprehensive analysis of kidney samples from patients with lupus nephritis (SLE, *n* = 19), ANCA-GN (*n* = 32) and anti-GBM disease (*n* = 6) and from healthy kidney tissue (Fig. 1a). All patients with RPGN were included in the Hamburg Glomerulonephritis Registry (Table 1). Gene expression profiling of 480 genes at high resolution was performed on tissue sections using an in situ multiplexed mRNA assay (10x Xenium)^9,11^. Kidney biopsies were distributed across eight Xenium slides (Fig. 1a). Samples from different diseases were distributed across the slides to minimize potential systematic variations (Extended Data Fig. 1a).

**Table 1:** Baseline characteristics

	ANCA-GN (*n* = 32)	SLE (*n* = 19)	Anti-GBM (*n* = 6)
Age, median (interquartile range)	55.5 (51–64.75)	35 (29–42)	57.5 (25.25–68.5)
Sex			
Female, *n* (%)	11 (34.37)	14 (73.68)	2 (33.33)
Male, *n* (%)	21 (65.63)	5 (26.32)	4 (66.67)
Histology, *n* (%)	ANCA renal risk score	Lupus nephritis class	Percentage crescentic or sclerotic glomeruli
	Low: 7 (24.14) Medium: 16 (55.17) High: 6 (20.69)	Class III: 7 (38.89) Class IV: 4 (22.22) Class III + V: 5 (27.78) Class IV + V: 2 (11.11)	<25: 0 <50: 0 <75: 2 (33.33) ≥75: 4 (66.67)
Laboratory values, median (interquartile range)			
Creatinine (mg dl^−1^)	2.5 (1.78–3.84)	0.9 (0.66–1.4)	2.0 (1.93–6.81)
eGFR (ml min^−1^)	25.79 (13.77–36.4)	89 (53–112)	14 (8.92–31.28)
Albumin-to-creatinine ratio (mg g^−1^)	1,165 (378–2,175)	1,100 (210–2,130)	1,140 (325–11,830)
Autoantibody levels (U ml^−1^)	Myeloperoxidase: 88 (55.59–122.3) Proteinase 3 antibody: 41 (1.47–145.3)	Double-stranded DNA: 55 (13–379)	Anti-GBM: 160 (29–765)

### Panel design to identify cells and disease progression

Our first step was to design a comprehensive gene panel for the identification of different kidney cells (Fig. 1b), immune cells and genes that may be important in glomerulonephritis at the molecular level. We started with a detailed literature review to identify cell-type-specific markers that would enable the identification of kidney and immune cells, including subtype populations^12–14^. Next, we included genes that are: (1) implicated in the pathogenesis of cGN; (2) essential for pathways involved in leukocyte infiltration and activation; (3) associated with cytokine and chemokine signaling and inflammation from the Kyoto Encyclopedia of Genes and Genomes^15^, Reactome^16^ and Gene Ontology^17^ databases; and (4) differentially expressed between normal and inflamed glomerular regions, based on our recently published spatial transcriptomics data from patients with ANCA-GN and controls^18^. These steps resulted in the selection of 480 genes that were used in this study (Supplementary Table 1).

### Segmentation and classification of kidney and immune cells

**Extended Data Fig. 2:**
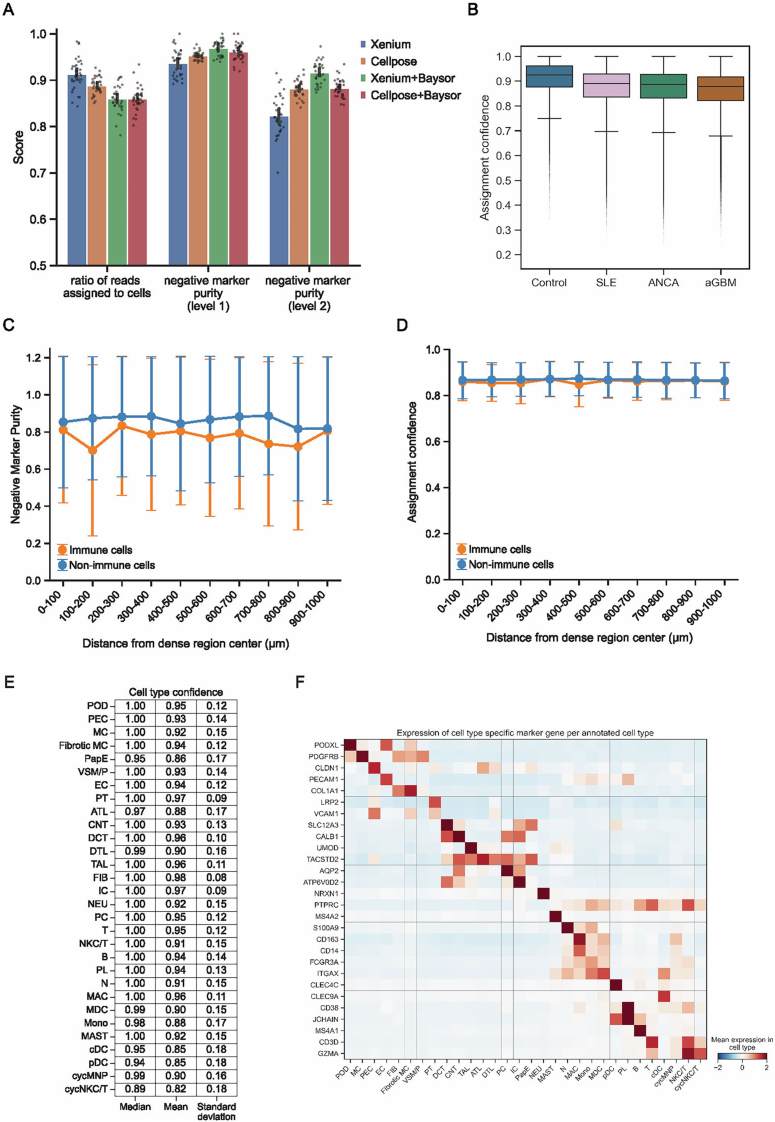
Evaluation of cell segmentation, classification and profiles. **a**, Bar plots showing comparison of segmentation quality metrics across different cell segmentation methods. Metrics include ratio of reads assigned to cells, negative marker purity at level 1 (all immune cells grouped), and negative marker purity at level 2 (with immune cell types as individual clusters). Bar graphs show mean and error bars represent the 95% confidence intervals. Each dot refers to the score of a biopsy (n=32, ANCA-GN biopsies). **b**, Boxplots showing transcript assignment confidence from Baysor across conditions (Control, SLE, ANCA-associated glomerulonephritis). Assignment confidence remains consistently high (~0.85-0.90) across all conditions, indicating robust transcript-to-cell assignments regardless of disease state. **c**, Line plots showing negative marker purities of immune and non-immune cells across increasing distances (presented as 9 bins between 0 and 1000 μm) from the center of dense immune infiltrates. Points represent mean values and error bars represent standard deviation. Cell counts per distance bin for immune cells are—0-100 μm: 111, 100-200 μm: 87, 200-300 μm: 114, 300-400 μm: 155, 400-500 μm: 189, 500-600 μm: 207, 600-700 μm: 227, 700-800 μm: 356, 800-900 μm: 444, 900-1000 μm: 279; for non-immune cells: 0-100 μm: 382, 100-200 μm: 747, 200-300 μm: 976, 300-400 μm: 1,292, 400-500 μm: 1,657, 500-600 μm: 1,943, 600-700 μm: 2,277, 700-800 μm: 2,766, 800-900 μm: 2,502, 900-1000 μm: 1,825. **d**, Line plots showing transcript assignment confidence for immune and non-immune cells across increasing distances similar to panel **c**. Points represent mean values and error bars represent standard deviation. Cell counts per distance bin are the same as in panel **c**. **e**, Matrix showing the classifier confidence (median, mean and standard deviation in prediction of the cell types. **f**, Heatmap showing the expression of cell type marker genes in annotated cell types.

To localize cells and determine their boundaries, we first segmented them using Baysor^19^, resulting in the identification of 3,218,210 filtered cells that passed our quality control (QC) metric, with between 303,732 and 501,421 cells in each slide (Extended Data Fig. 1b,c). The cells expressed a median of 24 genes (control: 14; SLE: 25; ANCA-GN: 26; and anti-GBM: 27) and 36 median transcripts (control: 21; SLE: 41; ANCA-GN: 39; anti-GBM: 41) (Extended Data Fig. 1d). The median segmented cell diameter was 7.7 μm^2^ (Extended Data Fig. 1e). We compared Baysor-based segmentation with competing approaches (Xenium’s default segmentation and Cellpose) and found that Baysor cell segmentation followed by Xenium’s default, expansion-based cell segmentation achieved the lowest signal of negative markers compared to Cellpose (Extended Data Fig. 2a). Transcript assignment confidence was comparable across disease conditions and was not substantially affected by tissue region density (Extended Data Fig. 2b–d). We detected transcripts of cell-type-specific marker genes in both control and diseased glomeruli (Fig. 1c). These included markers for podocytes (*PODXL*), mesangial cells (*PDGFRB*) and PECs (*CLDN1*). In the tubulointerstitial regions, we found transcripts specific to proximal tubule cells (*GPX3*) and collecting duct cells (*AQP2*). Glomerulonephritis samples additionally showed expression of collagen (*COL1A1*) and immune cell markers for monocytes or macrophages (*LYZ* and *CD163*) and T cells (*CD3D*, *CD3E* and *CD3G*) (Fig. 1c).

Subsequently, we used a logistic regression classifier to predict cell types with a median confidence >0.89 across cell types (Fig. 1d and Extended Data Fig. 2e). The cell types showed differential enrichment of cell-type-specific marker genes (Extended Data Fig. 2f) and exhibited some of the expected differential abundance across diseases, such as the loss of podocytes and the increase of innate and adaptive immune cells in RPGN versus healthy controls (Fig. 1e). Exemplary localization of cell types within control and ANCA-GN are presented in Fig. 1f, together with the corresponding hematoxylin and eosin (H&E)-stained images, highlighting that glomerular and periglomerular regions are affected by cellular changes in disease. In summary, segmentation and classification resulted in a very high-quality set of >3 million spatially resolved renal and immune cells.

### Defining and analyzing the glomerular and periglomerular niches

**Fig. 2:**
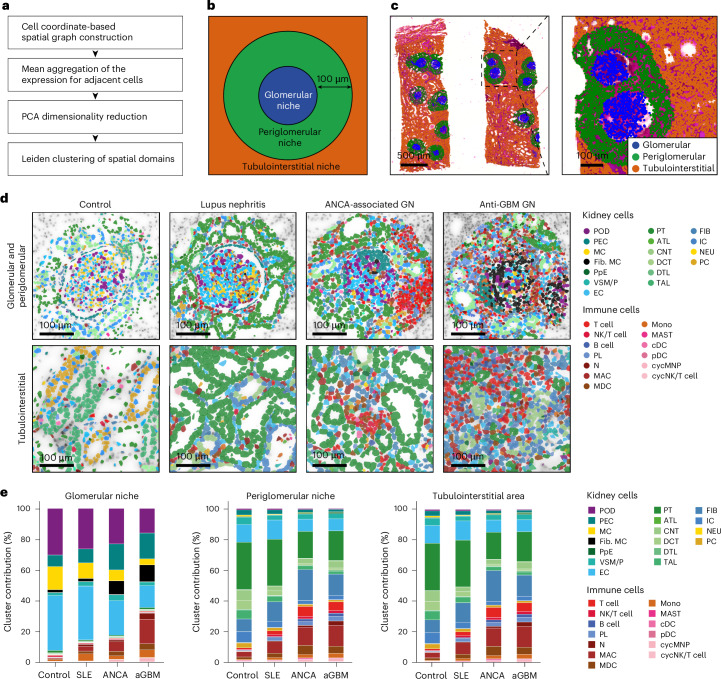
Spatial transcript analysis of renal compartments in glomerulonephritis. **a**, Workflow of the NichePCA algorithm used for automated annotation and spatial mapping of glomeruli. **b**, Schematic illustration of a glomerulus and its periglomerular niche, defined by expanding the glomerular boundaries to include surrounding regions. **c**, Representative H&E image showing glomeruli (blue) as defined by NichePCA, their corresponding periglomerular regions (green) and the remaining tubulointerstitial area (orange). **d**, Representative images highlighting the spatial localization of various kidney and immune cell types. The top panels show the spatial localization of various kidney and immune cells within the glomerular and periglomerular regions, whereas the bottom panels depict the same in tubulointerstitial regions across the four disease conditions. **e**, Stacked bar plots showing the proportions of different cell types across the four disease conditions within three spatial niches: the glomerular niche (left), periglomerular niche (center) and tubulointerstitial areas (right).

**Extended Data Fig. 3:**
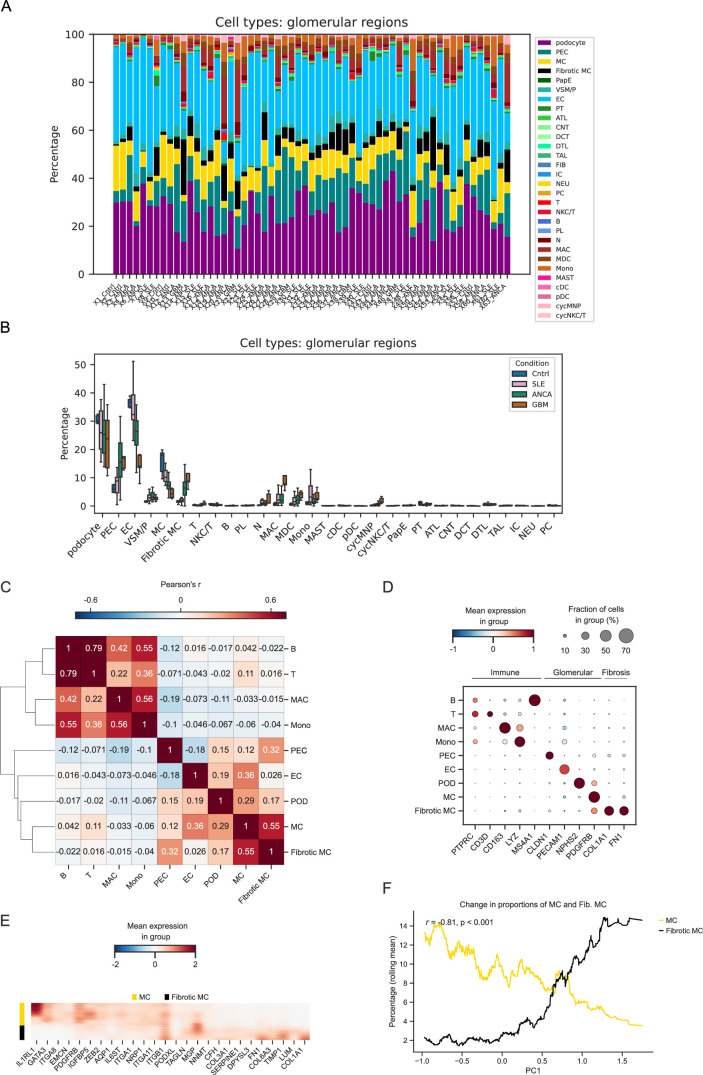
Cell type composition and analysis of glomerular regions. **a**, Stacked bar plot showing the cell type proportions for individual glomerular regions across control and disease conditions. **b**, Boxplots showing comparisons of the cell type proportions in glomerular regions between control, SLE, ANCA-GN, and anti-GBM conditions. The number of datapoints (cells per disease) used to create boxplots are—Cntrl: 29,035, SLE: 47,266, ANCA: 61,708, GBM: 31,744. Boxplots show the median (middle horizontal line), interquartile range (box), Tukey-style whiskers (lines beyond the box), outliers (data points beyond 1.5*interquartile or below −1.5*interquartile). **c**, Heatmap showing Pearson correlation coefficients between different ROI cell types (glomerular and abundant immune cell types) based on their transcriptional profiles. The heatmap displays strong positive correlations between MCs and fibrotic MCs (r = 0.55). **d**, Dot plot showing expression patterns of immune, glomerular, and fibrosis-related genes across cell types. Dot sizes represent the fraction of cells within the ROIs expressing each gene, and dot colors indicate scaled mean expression levels. **e**, Heatmap displaying the mean expression of selected genes in MC and fibrotic MC populations, highlighting transcriptional differences between these cell states. **f**, Line plot showing the change in proportions of MCs and fibrotic MCs ordered by PC1. The inverse relationship demonstrates potential transition from MC to fibrotic MC phenotype along this axis. Pearson’s correlation coefficient (r) and associated two-sided p-value (1.97e-174).

As, in glomerulonephritis, the glomerulus and the surrounding tubulointerstitial tissue might be most informative for understanding the immunopathogenesis of the disease (Fig. 1f), we focused on these areas. To obtain reliable annotation of the glomerular niches, we performed an automatic spatial domain annotation by employing NichePCA^20^ (Fig. 2a). The boundaries of the glomerular niches were subsequently extended by a 100-µm perimeter defining the periglomerular niches (Fig. 2b). We termed individual glomeruli and their associated periglomerular niche regions of interest (ROIs). Examples of identified glomerular and periglomerular niches are illustrated in Fig. 2c. Areas outside of glomerular and periglomerular niches were annotated as the tubulointerstitial domain. Figure 2d illustrates examples of these areas for each clinical condition, with cells colored by their annotated types. Control and, to some extent, SLE samples showed healthy glomerular structures with a monolayer of PECs around the glomeruli and low numbers of immune cells. In the ANCA-GN glomerulus, an increased number of PECs formed a crescentic structure inside the glomerulus. ANCA-GN and anti-GBM samples also showed increased accumulation of immune cells. A systematic comparison of the spatial accumulation of different cell types is shown in Fig. 2e and Extended Data Fig. 3a,b. In the glomerulus, podocytes constituted a median of 30% of cells in controls, which progressively decreased in SLE (25.9%) and ANCA-GN (25%) and was lowest in anti-GBM (23.8%) (Fig. 2e and Extended Data Fig. 3b).

In contrast, the percentage of PECs in the glomerulus exhibited the opposite trend, showing an increase in the disease conditions (control: 6%; SLE: 8%; ANCA-GN; and anti-GBM: 15%) (Fig. 2e and Extended Data Fig. 3b). In addition, there was a decrease in the percentage of mesangial cells (control: 17.9%; SLE: 10.2%; ANCA-GN: 7.1%; and anti-GBM: 4.3%) and a concomitant increase in fibrotic mesangial cells (control: 1.6%; SLE: 2%; ANCA-GN: 6.4%; and anti-GBM: 8.8%) in the glomerulus. The fibrotic mesangial cells display a distinct fibrotic signature, characterized by increased production of extracellular matrix (ECM) components such as collagens and fibronectin (Extended Data Fig. 3c–e). These cells have gene expression similar to mesangial cells (Extended Data Fig. 3c), but also express fibrosis-related genes such as *COL1A1* and *FN1* (Extended Data Fig. 3d,e) and their abundance is inversely correlated to that of mesangial cells (Extended Data Fig. 3f).

A decline in the proportion of glomerular endothelial cells (ECs) was also observed. Among immune cell types, a marked increase in innate immune cells was seen. Macrophages accounted for <0.5% of all glomerular cells in control samples which increased to 2% in SLE and ANCA-GN and 7.8% in anti-GBM (Fig. 2e and Extended Data Fig. 3b). Other innate immune cells were also increased within the glomerular niches. Notably, there was negligible increase in adaptive immune cells such as T cells, natural killer (NK) cells, B cells or plasma cells in these regions (Fig. 2e and Extended Data Fig. 3b).

**Extended Data Fig. 4:**
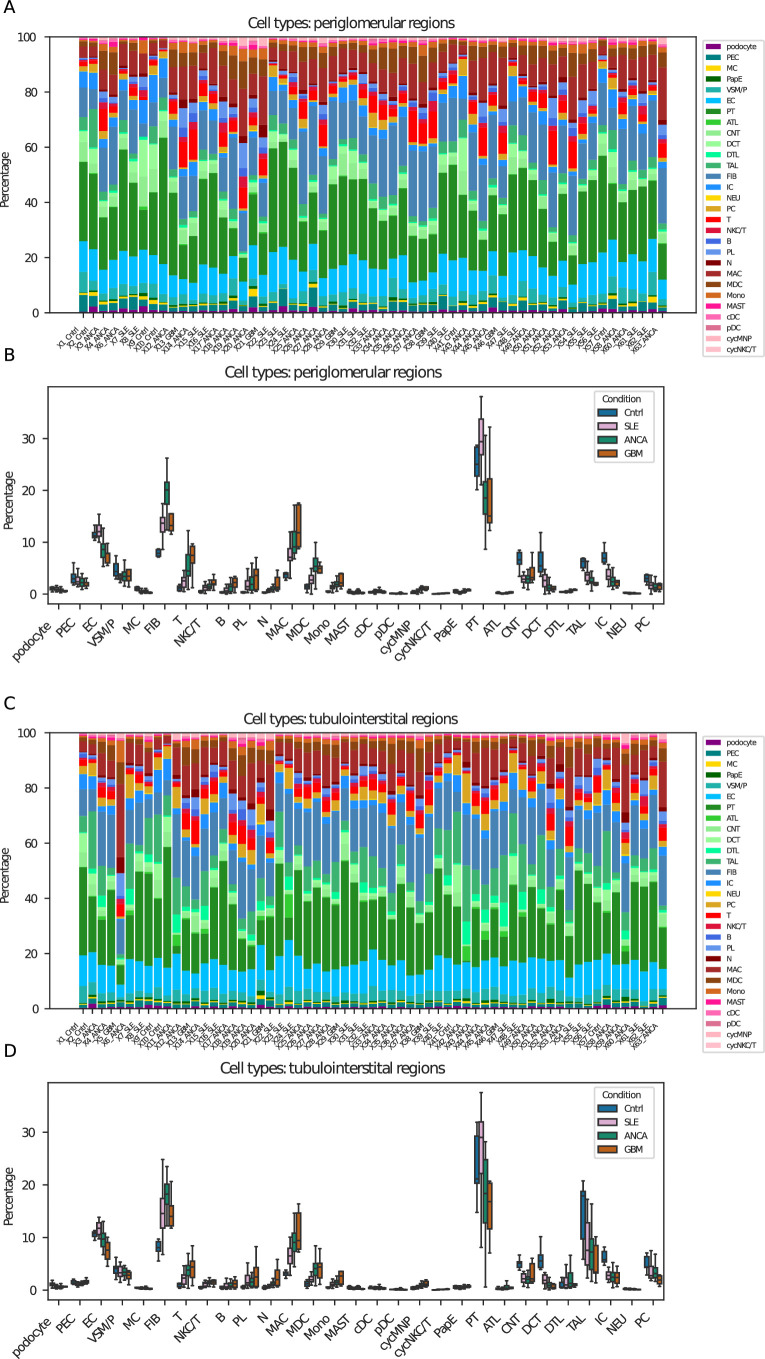
Cell type composition analysis of periglomerular and tubulointerstitial regions. **a, c** Stacked bar plot showing the cell type proportions for individual periglomerular regions (**a**) and tubulointerstitial regions (**c**) across control and disease conditions. **b, d** Boxplots comparing cell type proportions in periglomerular regions (**b**) and tubulointerstitial regions (**d**) between control, SLE, ANCA-GN, and anti-GBM conditions. The number of datapoints (cells per disease) used to create boxplots in panel **b** are—Cntrl: 59,045, SLE: 118,474, ANCA: 192,361, GBM: 66,024. The number of datapoints (cells per disease) used to create boxplots in panel **d** are Cntrl: 296,135; SLE: 632,488; ANCA: 1,318,963; GBM: 364,967. Boxplots show the median (middle horizontal line), interquartile range (box), Tukey-style whiskers (lines beyond the box), outliers (data points beyond 1.5*interquartile or below −1.5*interquartile).

By contrast, the periglomerular niches showed an increased accumulation of both innate and adaptive immune cells. T cells, which in control samples constitute about 1.1%, increased to a median of 2.5%, 4.8% and 7.4% in SLE, ANCA-GN and anti-GBM, respectively (Fig. 2e and Extended Data Fig. 4a,b). There was also a rise in the numbers of NK cells, B cells, plasma cells and several innate immune cells in the periglomerular area. In parallel with the periglomerular niches, tubulointerstitial regions showed enhanced infiltration by both innate and adaptive immune cells. T cells, for instance, increased from 1.13% in control samples to median proportions of 2.28% in SLE, 3.82% in ANCA-GN and 4.4% in anti-GBM (Extended Data Fig. 4c,d). In addition, NK cells, B cells, plasma cells, fibroblasts and other innate immune populations also increased in proportions in the tubulointerstitial regions (Extended Data Fig. 4d). Notably, we were not able to detect major differences between the periglomerular and remaining tubulointerstitial area (Extended Data Fig. 4).

Taken together, we observed an increased accumulation of immune and fibrotic cell types in all three diseases, whereas the spatial accumulation of the immune cells showed distinct characteristics. The glomerular compartment in these diseases exhibited a selective accumulation of innate immune cells, with a notable absence of adaptive immune cell infiltration. However, the periglomerular and tubulointerstitial domains exhibited increased innate and adaptive immune cells.

### Common trajectory of glomerular crescent formation

**Fig. 3:**
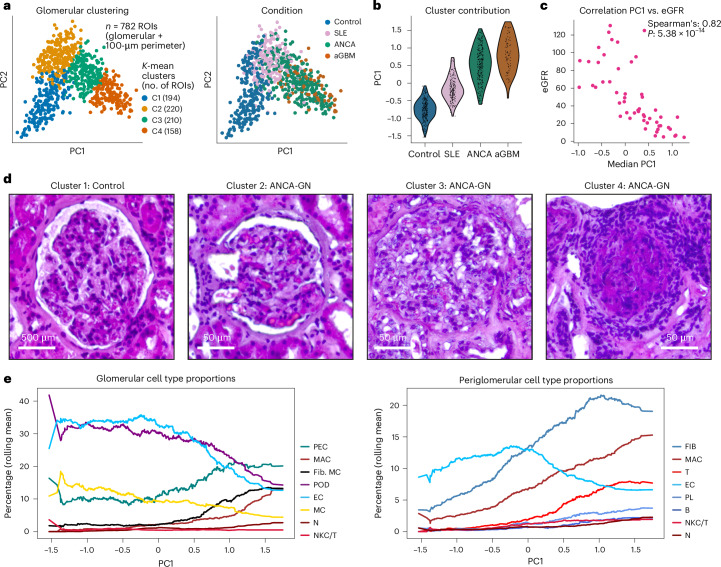
Trajectory of the immune cell ecosystem in glomerulonephritis. **a**, PCA plot showing 782 unique ROIs, each comprising a distinct glomerulus and its associated periglomerular area. ROIs are colored by clustering (left) and by disease condition (right). **b**, Distribution of PC1 values assigned to the ROIs across the four disease conditions. **c**, Correlation analysis of the median PC1 and kidney function (eGFR) of each sample (Spearman’s correlation, two-sided). **d**, Representative H&E images showing glomeruli from each of the four pseudotime quadrants, highlighting morphological variations: cluster 1: control (left); cluster 2: ANCA-GN (middle left); cluster 3: ANCA-GN (middle right); cluster 4: ANCA-GN (right). **e**, Change in the percentage distribution of different cell types within glomerular regions (left) and periglomerular regions (right) across the crescent formation trajectory (PC1).

**Extended Data Fig. 5:**
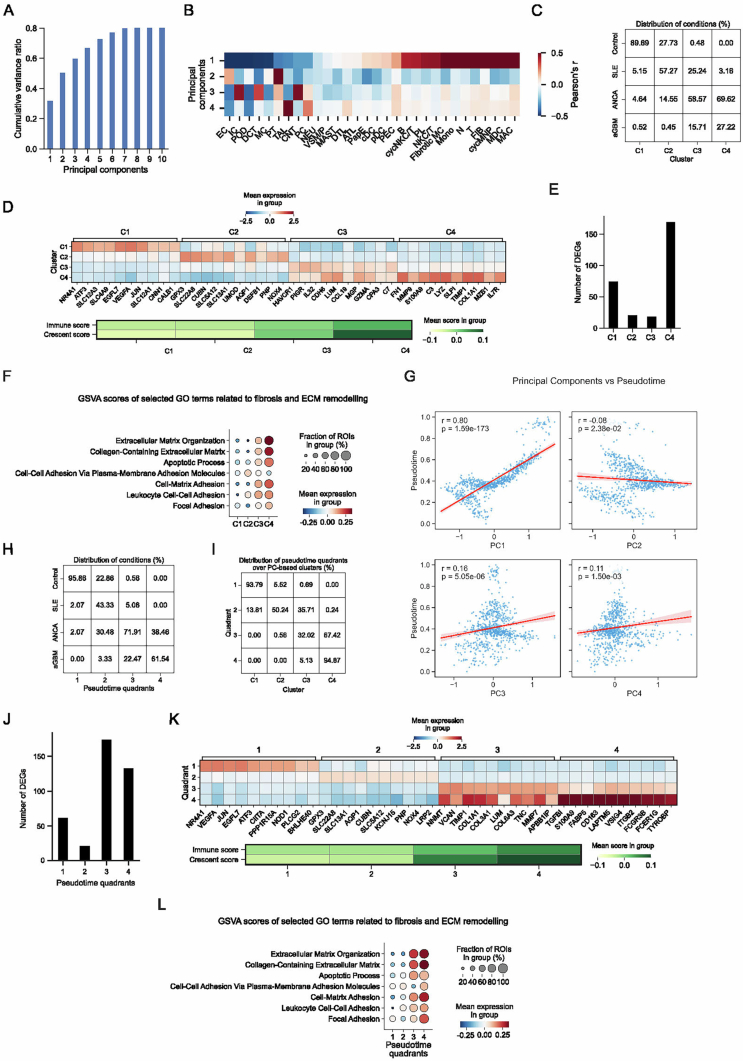
Principal component analysis, clustering of ROIs and pseudotime analysis. **a**, Cumulative variance ratio explained by the first 10 principal components. PC1 captures variance in the dataset. **b**, Heatmap showing Pearson correlation coefficients between cell type compositions and the first four principal components (PC1-4) across ROIs. Cell type composition was computed and correlated with its PC values. The cell types in the X-axis were ordered according to their correlation with the first principal component. **c**, Matrix showing the percentage distribution of conditions (Control, SLE, ANCA, and aGBM) across the four clusters. **d**, Heatmap showing the mean expression of top 10 markers across clusters C1-C4, with immune and crescent scores shown below. **e**, Bar plot showing the number of markers identified in each cluster. **f**, Dot plot showing enriched GO terms specifically related to fibrosis and extracellular matrix (ECM) remodeling across clusters. The dot size indicates the fraction of ROIs in each group, and colors represent GSVA scores for the enriched GO terms. **g**, Scatter plots showing linear associations between the first four principal components (PC1-4) and pseudotime for ROIs (Pearson correlation coefficients (r) and two-sided p-values). **h**, Matrix showing the percentage distribution of conditions (Control, SLE, ANCA, and aGBM) across the pseudotime quadrant. **i**, Matrix showing the percentage distribution of ROI clusters across the pseudotime quadrants. **j**, Bar plot showing the number of markers identified in each pseudotime quadrant. **k**, Heatmap displaying the mean expression of top 10 markers for each pseudotime quadrant, with immune and crescent scores shown below. **l**, Dotplot showing enriched GO terms specifically related to fibrosis and extracellular matrix (ECM) remodeling. The dot size indicates the fraction of ROIs in each group, and colors represent GSVA scores for the enriched GO terms.

As outlined in the previous section, the different diseases showed qualitatively similar cellular changes across the three compartments, albeit with differing median quantities. These findings suggest that the path to glomerulonephritis might be common across ANCA-GN, SLE and anti-GBM, whereas temporal stages and the speed of progression might be distinct. To find common trajectories and identify the molecular and cellular components of progression, we analyzed the trajectory of crescent formation based on a principal component analysis (PCA) of 782 combined unique ROIs (glomeruli plus periglomerular area) (Extended Data Fig. 5). The first principal component (PC1) of the ROIs showed a cellular signature of crescent formation, including positive correlations with immune cells, PECs and fibroblasts, while negatively correlating with podocytes and ECs (Extended Data Fig. 5a,b). In line, ROIs from controls displayed the lowest PC1 values and, from anti-GBM disease, the highest PC1 values (Fig. 3a,b).

To further understand the molecular and cellular characteristics of the spatiotemporal progression into crescents, we clustered the ROIs using PCs, revealing four clusters (C1–C4) containing 194, 220, 210 and 158 ROIs, respectively, each showing distinct distributions of conditions (Extended Data Fig. 5c). The first cluster was dominated by control ROIs (89.69%). The second cluster showed a mixed distribution, comprising control (27.73%), SLE (57.27%), ANCA-GN (14.55%) and anti-GBM (0.45%) ROIs. In contrast, the third and fourth clusters were primarily characterized by ANCA-GN and anti-GBM (Extended Data Fig. 5c). We compared the gene expression profiles of clusters to find marker genes. The ROI clusters showed different marker genes (C1: 75; C2: 21; C3: 19; and C4: 170) (Extended Data Fig. 5d,e). Among the top 10 genes enriched in clusters 3 and 4 were several immune-related cell type and fibrosis-associated genes (immune: *MZB1*, *LYZ* and *S100A9*; fibrosis: *FN1, LUM* and *TIMP1*)^21,22^. These two clusters also showed a higher enrichment of several terms related to fibrosis and ECM remodeling, with a higher enrichment in cluster 4 (Extended Data Fig. 5f). It is interesting that the results of the PCA closely resembled those of an additional diffusion pseudotime analysis, observing a significant correlation (Spearman’s *r* = 0.80) between PC1 and the pseudotime path (Extended Data Fig. 5g–l). This suggests that PC1 captures the common, progressive formation of crescents across the diseases, rather than the disease-specific progression time, which is partially captured in the other PCs.

**Extended Data Fig. 6:**
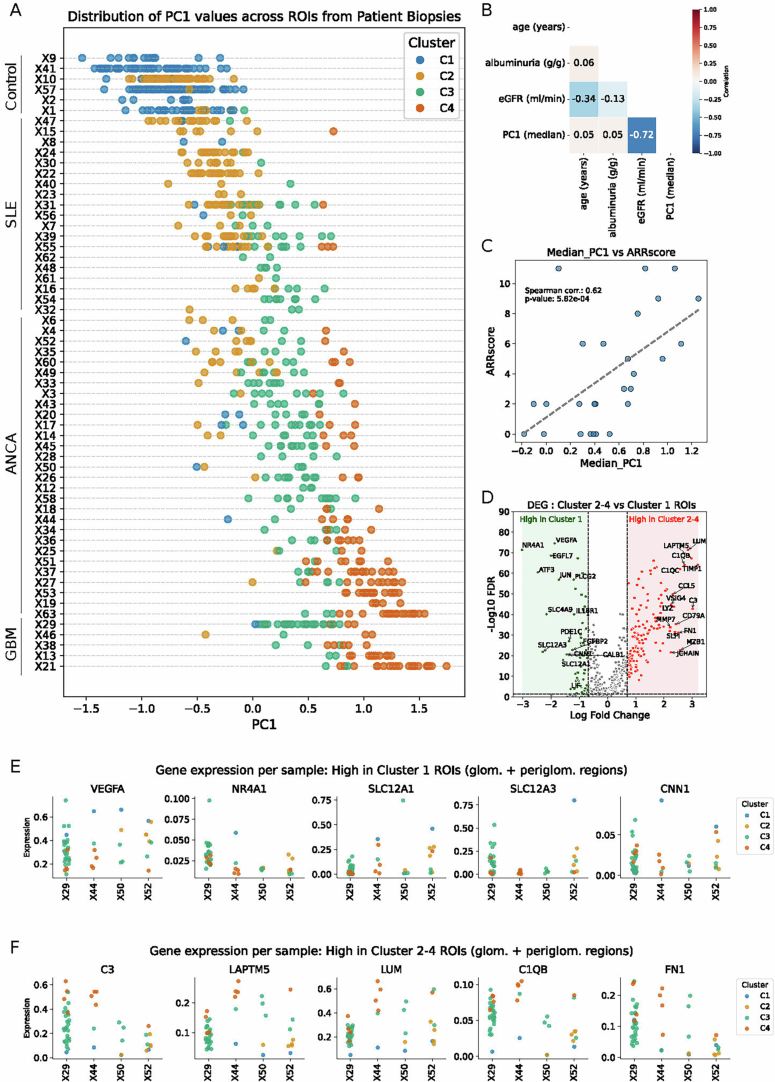
Profiling of renal biopsies of patients reveals PC1-associated transcriptional and clinical heterogeneity. **a**, Distribution of PC1 values across regions of interest (ROIs) from an individual patient’s biopsy. ROIs are color-coded by cluster identity and ordered along the Y-axis by increasing PC1 values and clinical condition. **b**, Correlation between the median PC1 value of ROIs from each patient and clinical variables: estimated glomerular filtration rate (eGFR), albuminuria and age (Pearson´s correlation, two-sided). **c**, Correlation between the ANCA Renal Risk (ARR) score of patients diagnosed with ANCA-GN and the median PC1 values of ROIs of their biopsy sample (Spearman’s correlation, two-sided). **d**, Differential gene expression analysis between ROIs classified as cluster 1 compared to those classified under clusters 2, 3 or 4. **e**, Expression of genes higher in cluster 1 ROIs shown in representative patients. **f**, Expression of genes higher in cluster 2-4 ROIs shown in representative patients.

Analysis of individual patient biopsies showed a large variation in the PC1 values of different ROIs from the same patient, several times spanning three clusters (Extended Data Fig. 6a). We computed the median PC1 value across all ROIs per patient and correlated that with other clinical data. The median PC1 value showed an inverse correlation with kidney function (estimated glomerular filtration rate (eGFR)) at the time of biopsy (Fig. 3c and Extended Data Fig. 6b). Among patients with ANCA-GN, the median PC1 values also positively correlated with the ANCA renal risk score, linking PC1 to clinical severity (Extended Data Fig. 6c). Differential gene expression analysis between ROIs in cluster 1 (preserved glomeruli) and those in clusters 2, 3 and 4 (more affected glomeruli) showed higher expression of inflammatory and fibrotic genes (for example, *C3*, *LUM* and *FN1*) in clusters 2–4. Conversely, genes associated with normal kidney function, such as *SLC12A1*, were expressed higher in cluster 1 ROIs (Extended Data Fig. 6d). The expression of these genes showed variation between ROIs within the same patient (Extended Data Fig. 6e,f). Examples of ROIs within each cluster are presented in Fig. 3d and indicate progressive glomerular pathology. The change in the percentage distribution of different cell types within the glomerular and periglomerular regions across the crescent formation trajectory (PC1) is shown in Fig. 3e.

These results indicate that SLE, ANCA-GN and anti-GBM share a common cellular and molecular trajectory of crescent formation, which might contain shared intercellular and intracellular signaling pathways. In this context, it needs to be pointed out that the results from anti-GBM samples (*n* = 6) have restricted power compared to SLE (*n* = 19) and ANCA-GN (*n* = 32). The common crescentic path dominated both the pseudotime and the PC-based analyses, but the data also underscore disease-specific temporal changes. However, these data are derived from a cross-sectional study and need to be distinguished from time course analysis, which could provide true information about actual temporal changes.

### Intercellular signaling of glomerular crescent formation

**Fig. 4:**
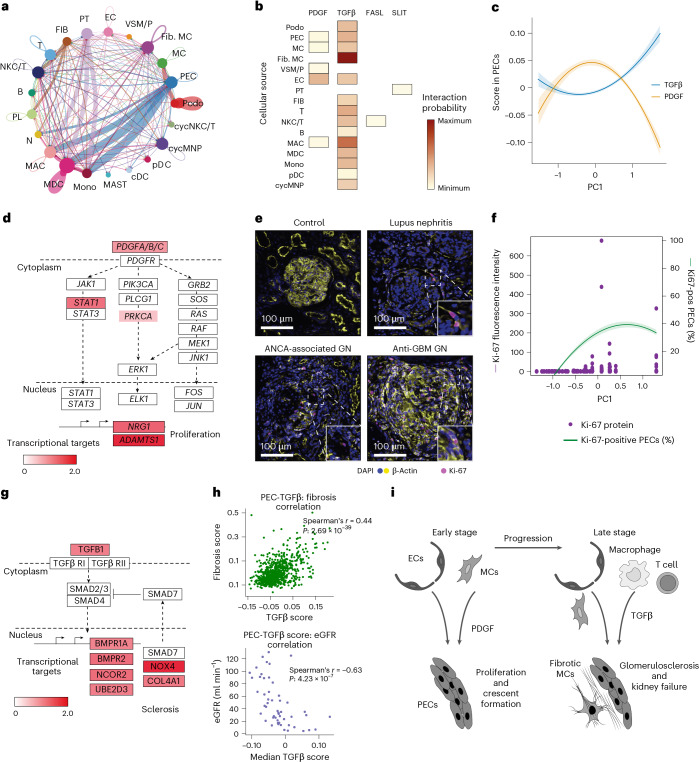
Mapping the immune–epithelial cell interactions in glomerular crescent formation. **a**, Circle plot showing cell–cell interactions in cGN compared to control samples. The interaction edges are colored the same as the source cell type whereas edge weights are proportional to the interaction strength. **b**, Heatmap showing the signaling pathways that have increased activity directed toward PECs in cGN and the source cell types producing the corresponding ligands, colored by the interaction probability. **c**, Scores for PDGF and TGFβ pathway activation in PECs plotted along pseudotime (lines show quadratic regression fits with 95% confidence intervals). **d**, Schematic representation of the PDGF signaling pathway, highlighting the genes upregulated in PECs in an snRNA-seq dataset from samples from patients with ANCA-GN (*n* = 11) over controls (*n* = 4). **e**, Multiplexed protein staining on a Xenium slide showing overexpression of Ki-67 in cGN samples (representative images of eight samples: two control (top left), three ANCA-GN (bottom left), two SLE (top right) and one anti-GBM disease (bottom right); representative of two independent experiments). **f**, Expression of Ki-67 protein in PECs along PC1. The primary axis corresponds to fluorescence intensity values of Ki-67. The secondary axis curve shows the change in percentage of Ki-67-positive cells per glomerulus. **g**, Schematic representation of the TGFβ signaling pathway, highlighting the genes from this pathway upregulated in PECs in an snRNA-seq dataset from samples from patients with ANCA-GN (*n* = 11) over controls (*n* = 4). **h**, Scatter plot showing correlation of TGFβ pathway score with fibrosis score in PECs (top) and the patient median TGFβ pathway score to the individual kidney function (bottom) (Spearman’s correlation, two-sided). **i**, Schematic illustrating the role of PDGF-mediated and TGFβ-mediated activation of PECs in crescent formation and glomerulosclerosis in cGN.

**Extended Data Fig. 7:**
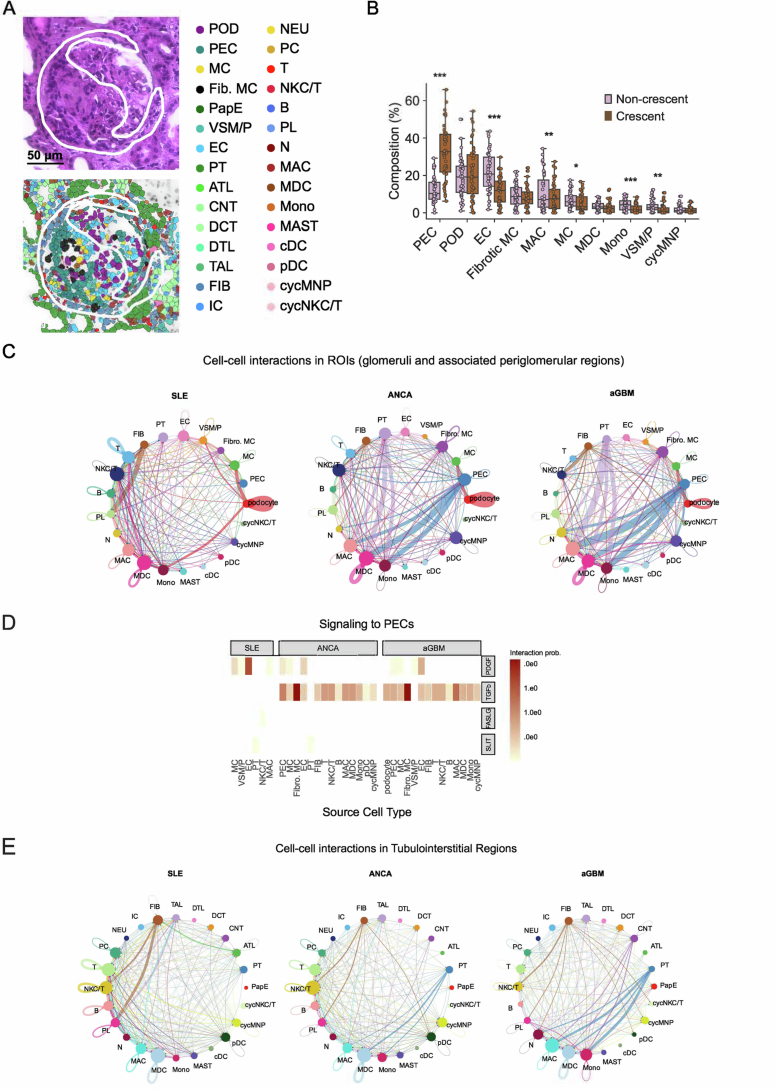
Crescent cell type composition and communication in ANCA-GN. **a**, Exemplified crescentic glomerulus annotated in white overlaid on DAPI staining (top panel) and corresponding cell type annotations (bottom panel). **b**. Box plots comparing cell type proportions between crescentic and non-crescentic glomerular regions (n=44). It shows the ten most abundant cell types in crescentic areas, ranked by average abundance (p-values for PEC: 6.2990e-12, EC: 8.3491e-10, MAC: 0.0066, MC: 0.02911; Mono: 0.0002, VSM/P : 0.0010). *p-value<=0.05, *p<=0.01, ***p<=0.001 (two-sided paired t-test comparing crescent and non-crescent regions of the same glomeruli. P-value correction was not performed. Boxplots show the median (middle horizontal line), interquartile range (box), Tukey-style whiskers (lines beyond the box), outliers (data points beyond 1.5*interquartile or below −1.5*interquartile). **c**, Circular network diagrams depicting intercellular communication patterns within the ROIs (glomerular+periglomerular regions) in SLE, ANCA-GN, and anti-GBM. Nodes represent different cell types and edges indicate communication strength between cell pairs, with edge thickness proportional to interaction probability. **d**, Heatmap showing disease-specific cell-type sources and their signaling pathways targeting PECs across SLE, ANCA-GN, and anti-GBM conditions. **e**, Intercellular communication patterns in the tubulointerstitial regions in SLE, ANCA-GN, and anti-GBM.

To elucidate the intercellular signaling pathways that underlie the RPGN development of glomerulonephritis, we performed interactome analyses using CellChat v.2^23^, which provides a framework for inferring cell interaction probabilities by integrating the ligand–receptor expression data. Importantly, CellChat v.2 uses the spatial proximity between cells and a maximum diffusion threshold of 250 µm (ref. ^23^). We focused on the regions of biggest change, the glomerular and periglomerular regions. In general, cGN samples were found to have a higher number of cell–cell interactions compared to control samples. A comparison of the cell-interaction networks between cGN and control samples revealed that PECs exhibited increased interactions with neighboring cell types in glomerulonephritis (Fig. 4a). In addition, analysis of affected glomeruli revealed a significantly higher proportion of PECs in the crescentic regions compared to noncrescentic regions (Extended Data Fig. 7a,b). Given the increased signaling activity originating from PECs, the high abundance of PECs in crescents and their potential central role in crescent formation, we investigated which pathways result in PEC activation. We filtered all significant cell–cell interactions in cGN and identified PDGF, TGFβ, FASL and SLIT pathways showing activity toward PECs (Fig. 4b). The primary source of PDGF ligands to PECs were ECs followed by mesangial cells, vascular smooth muscle cells or pericytes, PECs and macrophages. TGFβ was the other key signaling pathway targeting PECs, signaling from fibrotic mesangial cells, ECs, podocytes, PECs, fibroblasts and different immune cells (macrophages, monocytes, T cells, NK cytotoxic T cell (NKC/T) and B cells) (Fig. 4b). The interaction maps for the three cGN diseases separately showed similar results (Extended Data Fig. 7c,d). Cell interaction analysis of the tubulointerstitial regions revealed increased signaling from proximal tubules and fibroblasts to different immune cell types (Extended Data Fig. 7e).

### PDGF induces PEC proliferation and crescent formation

We next investigated the dynamics and effects of early PDGF activation in PECs in glomerulonephritis. Therefore, we quantified the activation levels of the PDGF pathway in PECs by creating a pathway-specific score. We used the Reactome gene set for PDGFR signaling in disease (curated gene set collection (C2) from the Molecular Signatures Database, MSigDB)^24^. We plotted this PDGF receptor (PDGFR) score in PECs against the crescent trajectory (PC1) from our analysis (Fig. 4c). The PDGFR score exhibited an increase at early to medium progression stages and decreased at advanced stages.

**Extended Data Fig. 8:**
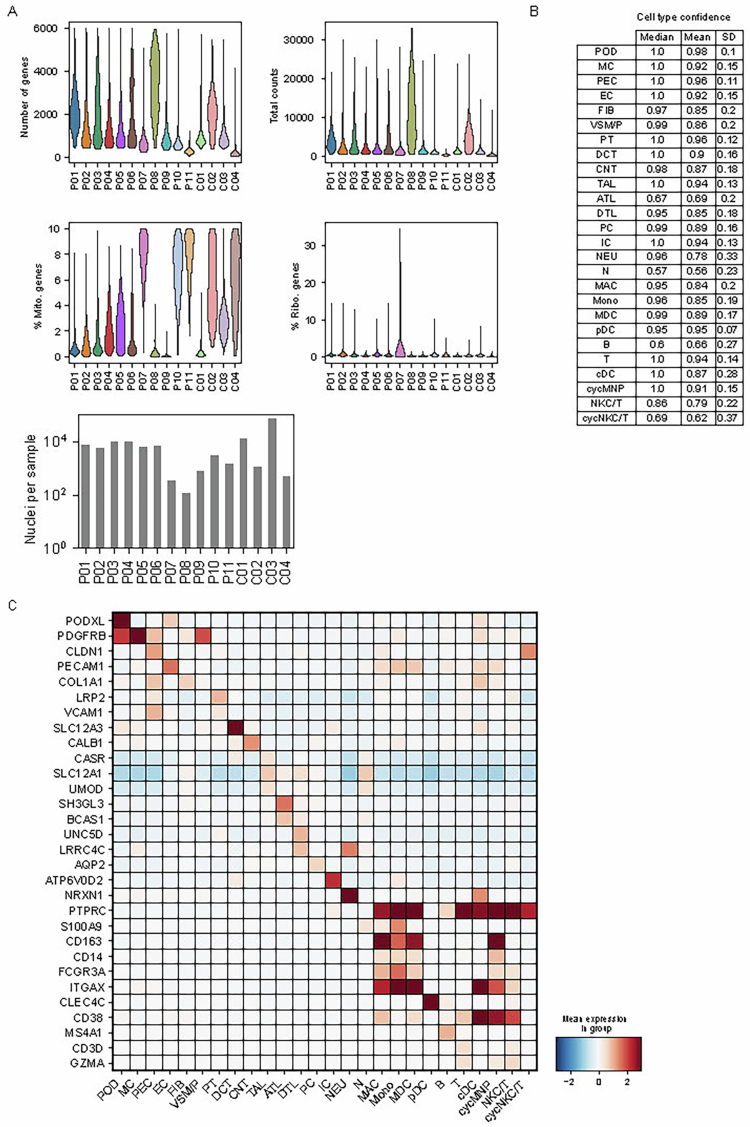
Single nuclear mRNA sequencing (snRNA-seq) QC and cell type annotation. **a**, Violin plots show quality control metrics, distribution of number of genes, total counts, percentage of mitochondrial and ribosomal genes across samples. Barplots show the total number of nuclei per sample. **b**, Matrix showing the classifier confidence (median, mean and standard deviation in prediction of the cell types. **c**, Heatmap showing the expression of cell type marker genes in annotated cell types (Wilcoxon test, adjusted p-value <0.05).

To validate the activation of PDGF pathways observed in PECs within the spatial transcriptomics dataset, using an independent orthogonal dataset with higher transcript resolution per cell, we performed single nuclear transcriptome sequencing (single nuclear RNA sequencing (snRNA-seq)) from kidney biopsies of patients with ANCA-GN (*n* = 11) and controls (*n* = 4), resulting in 136,376 nuclei after QC filtering (Extended Data Fig. 8a). Cell types were predicted using a logistic regression classifier (Extended Data Fig. 8b,c). Genes associated with the PDGF pathway were found to be upregulated in PECs from ANCA-GN compared to controls, further confirming the activation of these pathways in ANCA-GN (Fig. 4d). Genes transcriptionally upregulated downstream of PDGF included those involved in regulating proliferation, such as *NRG1* (ref. ^25^) and genes associated with fibrosis such as *ADAMTS1* (ref. ^26^) (Fig. 4d).

To investigate whether high PDGF signaling results in proliferation of PECs, we investigated the presence of Ki-67 protein along the crescent trajectory (PC1). To this end, we combined Xenium transcript analysis with Akoya PhenoCycler-based spatial proteomics of the same slide for eight samples (two controls, three ANCA-GN, two SLE and one anti-GBM disease; Fig. 4e). To align spatial transcriptomic and proteomic images, we registered the Akoya PhenoCycler and Xenium DAPI signals using VALIS^27^. Ki-67 intensity levels were assigned to PECs within all ROIs based on the cell boundaries obtained by our cell segmentation. Thus, we were able to identify PECs based on their transcriptional profile, align glomeruli according to their PC1 trajectory and determine proliferation by Ki-67 quantification. These analyses confirmed that PEC proliferation followed similar dynamics (Fig. 4f) to PDGF signaling in PECs (Fig. 4c).

### TGFβ signaling results in glomerulosclerosis

Next, we determined the functional effects of TGFβ signaling on PECs in advanced crescents. We used the human gene set of the TGFβ signaling signature (Hallmark gene set collection H from MSigDB^24^). The TGFβ score showed a continuous increase over the crescent trajectory (PC1) (Fig. 4c). For TGFβ, snRNA-seq of differentially expressed genes from ANCA-GN versus control PECs revealed upregulation of the TGFβ pathway genes that play crucial roles in fibrosis (*COL4A1* and *NOX4*) (Fig. 4g). *COL4A1* encodes type IV collagen, a key component of the ECM that contributes to ECM stiffening and tissue remodeling^28^. NOX4 is a major source of reactive oxygen species and promotes oxidative stress, driving fibrosis and ECM deposition^29,30^. The role of NOX4 in renal fibrosis is complex and has also been shown to have a protective role^31^. Other genes activated by the TGFβ pathway are *BMPR1A* and *BMPR2*, which code for receptors for BMP4, a ligand within the TGFβ superfamily, and *NCOR2*, coding for a transcriptional corepressor that modulates fibrosis^32^.

Finally, we aimed at understanding whether TGFβ signaling in PECs could indeed result in glomerulosclerosis in human glomerulonephritis. The TGFβ signaling score correlated with a fibrosis score (Fig. 4h, upper panel) and inversely correlated with kidney function (eGFR) (Fig. 4h, lower panel). These findings support the hypothesis that TGFβ signaling is activated in RPGN and contributes to glomerulosclerosis and kidney failure.

**Fig. 5:**
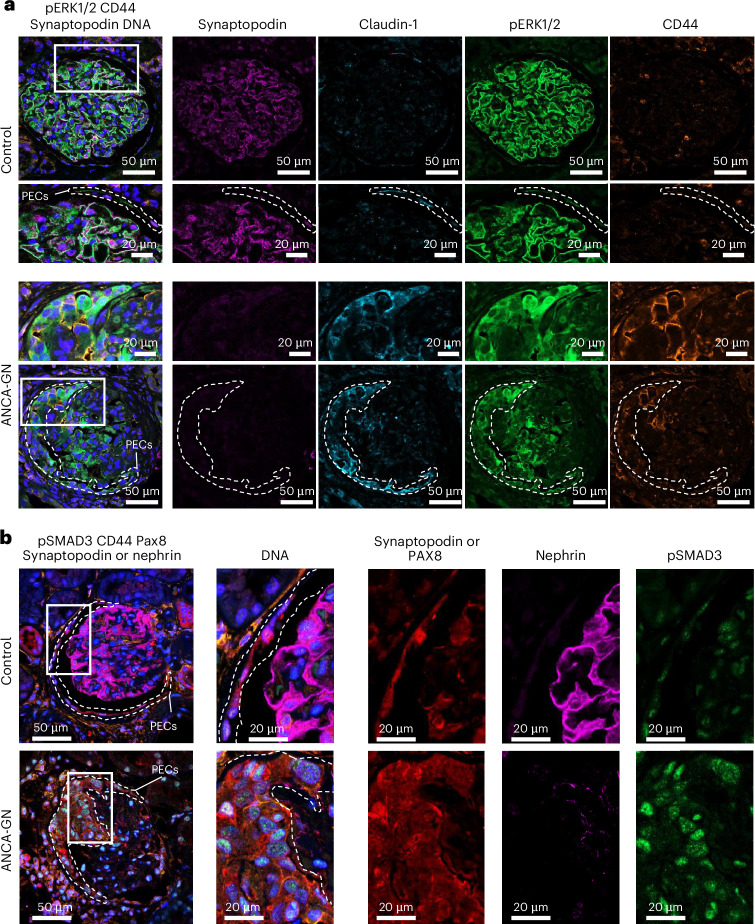
Immunohistochemistry of PECs and intracellular signaling molecules. **a**, Exemplified presentation of immunofluorescence staining showing prominent phospho-ERK1/2 and CD44 positivity in claudin-1^+^ or synaptopodin^−^ PECs in human kidney biopsies of patients with ANCA-GN (bottom) compared to healthy control tissue (top). **b**, Exemplified presentation of immunofluorescence staining showing prominent phospho-SMAD3 staining in PAX8^+^ or synaptopodin^−^ PECs in human kidney biopsies of patients with ANCA-GN (bottom) compared to healthy control tissue (top) in exemplified immunofluorescence staining. Representative staining of four controls and four patients with ANCA-GN is shown.

To further investigate the activation of PECs in RPGN, we performed immunofluorescence staining of human control samples and kidney samples of patients with ANCA-GN. PECs were defined as claudin-1^+^ and synaptopodin^−^. These analyses confirmed prominent MAPK signaling (pERK1/2) in PECs from patients with ANCA-GN and PEC activation (CD44) compared to control PECs (Fig. 5a). These findings indicate that PDGF signaling acts on PECs and induces cell activation and proliferation in patients with RPGN. Immunofluorescence staining confirmed activation of the TGFβ signaling pathway by prominent SMAD3 phosphorylation and nuclear expression in PECs of patients with ANCA-GN (Fig. 5b).

**Extended Data Fig. 9:**
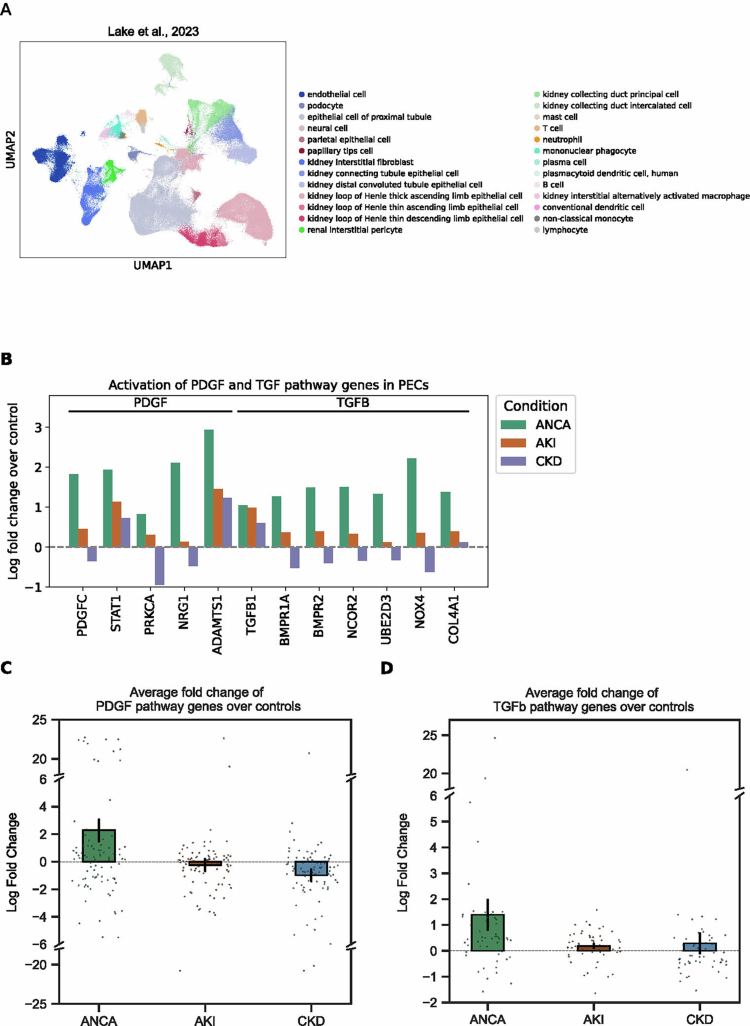
Comparison of PDGF and TGF-β pathway gene activation in PECs in crescentic glomerulonephritis (cGN) versus non-immune-mediated kidney diseases. **a**, UMAP showing major kidney cell types identified in the scRNA-seq dataset from Lake et al. (Nature, 2023), based on biopsies from patients with kidney diseases such as diabetic nephropathy or hypertension. **b**, Fold change in expression of PDGF and TGF-β pathway genes in PECs from ANCA patients versus controls (as also shown in Fig. 4d,g), compared to the log fold change observed in PECs from AKI or CKD patients relative to controls. **c–d**, Barplots showing average fold change of all genes used in the PDGF (C, n=89 genes)) and TGF-β (D, n=54 genes) pathway score calculations (barplots show mean ± standard error. The y-axes are split to show all data points).

To assess the generalizability of our findings, we analyzed publicly available single-cell (sc)RNA-seq or snRNA-seq data from the Kidney Precision Medicine Project, which includes biopsies from patients with nonimmune-mediated kidney diseases^13^ (Extended Data Fig. 9a). We found that PDGF and TGFβ pathway activation in PECs from patients with ANCA-GN in our snRNA-seq dataset was substantially higher than that seen in acute kidney injury or chronic kidney disease compared to respective controls (Extended Data Fig. 9b–d).

### Functional role of PDGF and TGFβ signaling in experimental GN

The analysis in human RPGN highlights that, first, PDGF induces cell proliferation of PECs and crescent formation and, later, immune and kidney cell-derived TGFβ results in PEC-mediated and fibrotic, mesangial cell-mediated glomerulosclerosis (Fig. 4i).

Next, we aimed to identify potential treatment options for targeting PDGF and TGFβ signaling. We therefore performed digital pharmacology^18^. Specifically, we calculated TGFβ and PDGF pathway scores and matched upregulated pathway genes against DrugBank^33^ to identify drugs targeting these pathways, prioritizing inhibitors. We identified nintedanib^34,35^ with the highest score for blocking PDGF signaling in PECs. In addition, fostamatinib was identified as a potential blocker of the TGFβ pathway. Although primarily targeting SYK, fostamatinib also exhibits off-target inhibition of TGFβ receptor 1 (ref. ^36^).

**Fig. 6:**
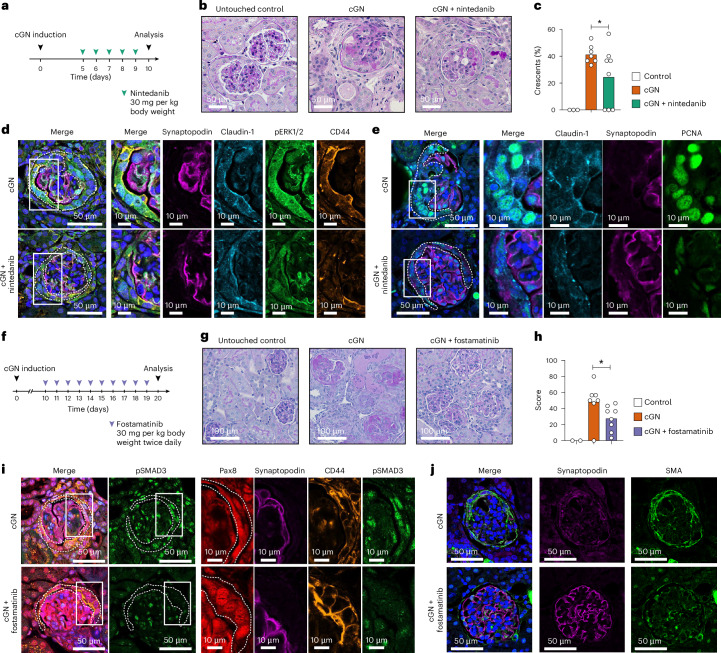
PDGF and TGFβ blockade in experimental crescentic glomerulonephritis. **a**, Experimental setup of PDGF blockade with nintedanib in experimental cGN. **b**, Periodic acid–Schiff staining of kidney sections of the respective groups. **c**, Quantification showing reduction of crescents in mice treated with nintedanib (data pooled from two independent experiments): left, untouched; middle, cGN; right, cGN + nintedanib. **d**, Exemplified presentation of immunofluorescence staining showing prominent phospho-ERK1/2 and CD44 positivity in claudin-1^+^ or synaptopodin^−^ PECs from cGN mice treated with nintedanib (bottom) in comparison to PBS (top). **e**, Low expression of the proliferation marker PCNA in claudin-1^+^ or synaptopodin^−^ PECs in exemplified immunofluorescence staining of cGN mice treated with nintedanib (bottom) in comparison to phosphate-buffered saline (PBS; top). **f**, Experimental setup of TGFβ blockade with fostamatinib in experimental cGN. **g**, Periodic acid–Schiff staining of kidney sections of the respective groups: left, untouched control; middle, cGN; right, cGN + fostamatinib. **h**, Quantification showing reduction of glomerulosclerosis in mice treated with fostamatinib (data pooled from two independent experiments). **i**, Exemplified presentation of immunofluorescence staining showing less prominent phospho-SMAD3 positivity in PAX8^+^ or synaptopodin^−^ PECs. **j**, Glomerular SMA staining in kidney sections of mice with fostamatinib-treated cGN (bottom) and cGN controls (top). One-sided Student’s *t*-test was performed (**c** and **h**). ^*^P < 0.05).

For functional validation of these findings, we used a well-established mouse model of cGN (also referred to as nephrotoxic nephritis)^37^. To block the PDGF pathway, after induction of cGN, mice were treated with nintedanib (oral (p.o.)) from day 5 to day 9 (Fig. 6a). At day 10, renal histology showed reduced crescents (Fig. 6b,c), accompanied by reduced ERK1/2 phosphorylation and decreased activation and proliferation of PECs (Fig. 6d,e).

To investigate TGFβ blocking at later stages, mice were treated with fostamatinib (intraperitoneally (i.p.)) from day 10 to day 19 (Fig. 6f). In mice treated with fostamatinib, glomerulosclerosis was reduced at day 20 (Fig. 6g,h). In addition, PECs displayed reduced SMAD3 phosphorylation (Fig. 6i) and glomerulosclerosis, as visualized by less prominent smooth muscle actin (SMA) staining (Fig. 6j).

These functional experiments in a mouse model support the findings from humans that PDGF, in the early stages of crescent formation, and TGFβ, in advanced stages, promote PEC activation and proliferation as well as glomerulosclerosis, respectively.

## Discussion

RPGN is characterized by immune cell infiltration into the kidney and crescent formation. The lack of knowledge about the immune and kidney cells, as well as their interactions, leading to glomerular damage and disease progression, has hindered the development of targeted therapies. Here we report a spatial high-resolution transcriptomic atlas of kidney biopsies from patients with RPGN, deciphering the cell composition and their communications involved in the sequence of crescent formation.

RPGN is a group of severe, rapidly progressive, autoimmune diseases marked by the formation of cellular crescents in Bowman’s space, leading to severe glomerular injury and loss of kidney function. The most common causes of RPGN include ANCA-GN, SLE and anti-GBM disease, in which the pathology is triggered by an intense inflammatory reaction within the glomerulus and often the periglomerular region^38^. Experimental GN models demonstrated that the recruitment of immune cells and the production of various inflammatory mediators^1^ lead to the rupture of glomerular capillaries and the activation and proliferation of PECs, which together with infiltrated leukocytes form the characteristic glomerular crescent^39–42^.

Recent single-cell and spatial transcriptome analyses in renal biopsy samples improved our understanding of kidney immunity in patients with RPGN by identifying inflamed regions, leukocytes and predominant cytokine signaling in the tissue^18,43,44^. However, identification of immune cell subtypes and their precise localization in glomerular and periglomerular regions has so far not been possible due to the low resolution of the spatial approach^18^. By using high-resolution spatial transcriptomics technologies^9,10^, we can precisely define immune and kidney cells within the glomerular, periglomerular and interstitial regions in a large cohort of patients with RPGN. We observed a notable enrichment of innate immune cells such as monocyte-derived cells and macrophages within the glomeruli in all three cGN subtypes. In contrast, adaptive immune cells were predominantly found in the periglomerular regions.

In addition, our investigation of cell–cell interactions in the glomerular and periglomerular compartments highlighted increased activation of PECs, driven by PDGF and TGFβ signaling, primarily from ECs, mesangial cells, vascular smooth muscle cells or pericytes and various immune cell types. Trajectory analysis indicated that PDGF pathway activation is the driving force for PEC proliferation and crescent formation in the ‘inflammatory’ phase of cGN. By combining our spatial transcriptomic data with whole-kidney snRNA-seq data and additional immunofluorescence staining, we specifically showed PDGF signaling results in PEC proliferation.

The PDGF family comprises four ligand isoforms (PDGF-A, PDGF-B, PDGF-C and PDGF-D) and two receptor chains, (PDGFR-α and PDGFR-β), which are either constitutively or inducibly expressed^45^. Both PDGFR-α and PDGFR-β are consistently expressed by mesangial cells, fibroblasts and vascular smooth muscle cells, although notably absent in epithelial cells, such as visceral epithelial cells (podocytes) and tubular cells. However, a localized expression of PDGFR-β has been documented in PECs^46^. In the context of kidney diseases, de novo expression or upregulation of PDGF isoforms and their receptors has been reported in rodent models of renal injury, as well as in corresponding human renal pathologies. Binding of ligands to PDGF receptors induces autophosphorylation within the cytoplasmic tyrosine kinase domain of the receptor, recruitment of adapter proteins with SH2 and SH3 domains and activation of downstream signaling, including the JAK–STAT, PI3K–AKT, PLC-γ and RAS–MAPK pathways, which induce proliferation, migration and fibrosis^45^.

The factors that promote the transition from the inflammatory phase of cGN, defined by cellular crescents (which can be reversible), to an irreversible fibrotic phase with fibrous crescents and glomerulosclerosis is largely unknown. Our finding that TGFβ downstream signaling in PECs is predominantly detectable at later stages is therefore of interest. TGFβ is known as a central driver of tissue fibrosis, promoting ECM protein synthesis, inhibiting matrix degradation and inducing cell transformations that lead to glomerulosclerosis and tubulointerstitial fibrosis. Elevated TGFβ levels are strongly associated with progression in cGN^47,48^. TGFβ induces fibrosis through both SMAD-dependent and SMAD-independent pathways. TGFβ activation of fibroblasts and epithelial-to-mesenchymal transition (EMT) further amplifies fibrotic processes^49^. Various approaches to inhibit TGFβ have been explored, including neutralizing antibodies, receptor inhibitors, small interfering RNA and small molecule inhibitors. Clinical trials with anti-TGFβ agents, such as LY2382770 and fresolimumab, in fibrotic kidney diseases have yielded mixed results^50^. The failure of a drug response is attributed to the pleiotropic nature of TGFβ in both immune regulation and kidney fibrosis. However, our data suggest that TGFβ plays a more dominant role in the later stages of disease progression, so interventions at this stage may be more difficult to achieve therapeutic benefit.

**Extended Data Fig. 10:**
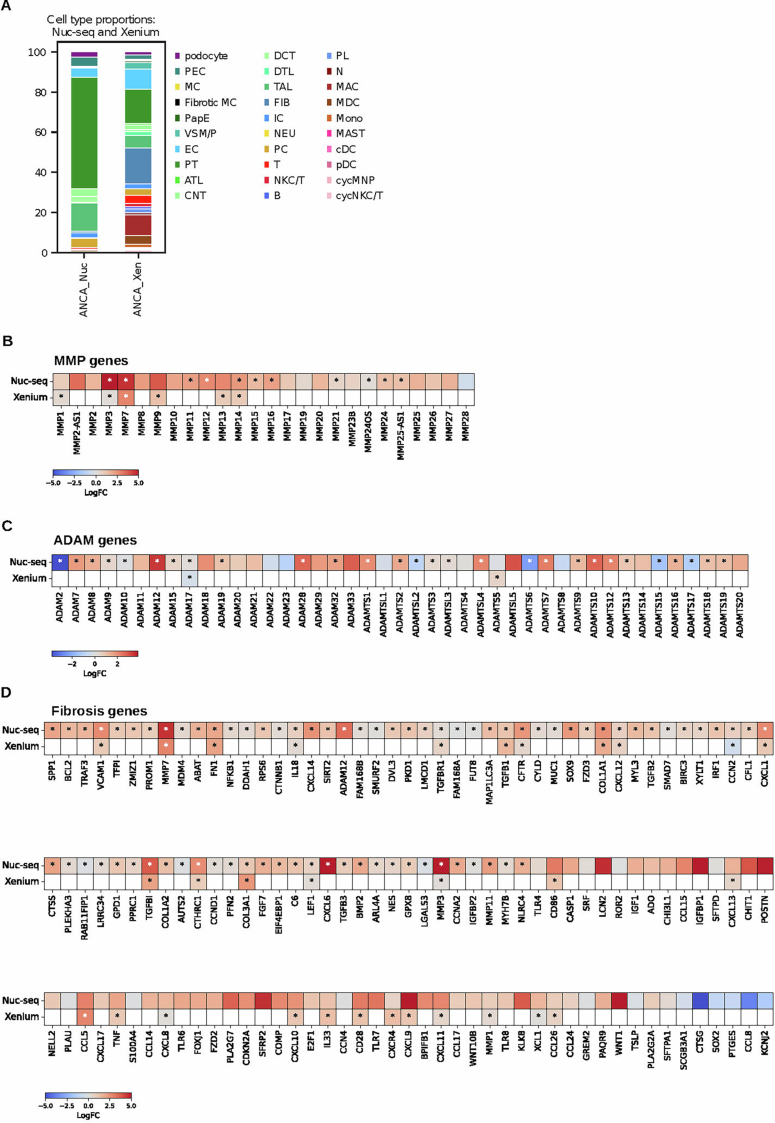
Comparison of cell type proportions and differential gene expression in ANCA-associated biopsies using single-nucleus RNA sequencing and Xenium spatial transcriptomics. **a**, Comparison of cell type proportions in biopsies from ANCA patients between NucSeq and Xenium datasets. **b–d**, Log fold change in expression of specific gene groups between ANCA and control samples, as measured by NucSeq and Xenium: MMP genes (**b**), ADAM genes (**c**), and fibrosis-related genes (**d**). The fibrosis related genes for human kidney were taken from Fibrotic Disease-associated RNAome database (http://www.medsysbio.org/FDRdb). * show p-value < 0.05, two-sided Wilcoxon test with Benjamini-Hochberg correction.

The spatial data generated in this study provided a valuable resource for studying the molecular details of human cGN. However, its scope is limited by the requirement of a predefined panel of 480 genes that was possible at that time. Although this panel was carefully designed to include key immune and kidney cell markers, as well as several genes implicated in cGN pathogenesis, it is not transcriptome wide. To address this limitation, we complemented the spatial transcriptomic data with an independent snRNA-seq dataset. This allowed for broader, transcriptome-wide profiling and studying genes that were absent in the spatial panel. Comparison of cell-type proportions between the snRNA-seq and Xenium datasets revealed that the Xenium data showed less bias toward overrepresentation of tubular cells, which may be a consequence of uneven recovery of viable cells of different types in scRNA-seq or snRNA-seq protocols. Instead, the spatial dataset could capture higher relative abundance of immune cells (Extended Data Fig. 10a), which makes it particularly useful for investigating smaller, yet pathologically and therapeutically important, cell populations. Differential gene expression analysis across the two datasets revealed similar upregulation of several genes in ANCA-associated GN biopsies, including matrix metalloproteinases and members of the ADAM family involved in TGFβ activation and fibrosis (Extended Data Fig. 10b,c). Furthermore, a broader set of fibrosis-associated genes was elevated in ANCA-GN samples (Extended Data Fig. 10d), reinforcing and extending the profibrotic signature identified in the spatial transcriptomic data.

The limitation of the restricted panel also holds true for the cell–cell interaction analysis that could not cover all possible intercellular communications. An additional challenge of the technique includes difficulty in cell-type annotation due to transcript spillover. During sample preparation, mRNAs could be released from their original cellular location, resulting in potential spillover into neighboring cells. To avoid this, we performed a very restricted cell separation. Apart from this, the clinical samples that are the basis for this analysis are cross-sectional and we could not provide an actual time course of events in crescent formation. Moreover, the size of the study was too small to correlate different treatment regimens or disease duration to the crescent formation trajectory. However, the trajectory analysis could be used to generate a framework of events resulting in glomerular deterioration. Finally, experimental GN models targeting PDGF and TGFβ signaling pathways specifically in PECs are of interest for future studies.

This study represents a large, spatial, high-resolution, multiplexed mRNA analysis dataset in combination with protein measurements, which was instrumental in decoding the sequential events in compartment-specific cellular composition and interaction, and resulted in the identification of new therapeutic targets in GN. This dataset can be harnessed by the scientific community for further exploration beyond this study. This technical approach to understand the immune and tissue response can act as a role model for future studies even in other autoimmune diseases.

## Methods

### Study design and patient inclusion

This study was designed to analyze immune cells and parenchymal kidney cells as well as transcriptional profiles of inflamed glomeruli in the kidney tissue of patients with rapidly progressive GN and in homeostatic kidney tissue. All patients with glomerulonephritis were included in the Hamburg Glomerulonephritis Registry. Healthy kidney tissue was obtained from unaffected kidney parts of tumor nephrectomies. All patients provided informed consent in accordance with the Consensus-based CAse REport (CARE) guidelines and the ethical principles stated in the Declaration of Helsinki. Information about the sex of the patients is provided in Table 1. Biological sex and self-reported sex were identical. Sex-based and gender-based analyses were not performed in this study. This study was approved by the institutional reviewing boards of the University Medical Center Hamburg-Eppendorf and the Ethik-Kommission der Ärztekammer Hamburg (local ethics committee of the chamber of physicians in Hamburg).

### High-dimensional spatial data generation

For performing spatial analysis, human kidney samples from fresh-frozen, paraffin-embedded tissue were used. The 5-µm-thick tissue sections were placed on Xenium slides and processed according to the manufacturer’s instructions (10x Genomics). Subsequently, H&E staining was performed and whole-slide images were obtained on a Leica DMi8 system, using the Leica Application Suite X (v.3.7.4.23463) software^18^. In the case of protein analysis, protein staining and image acquisition were performed on a PhenoCycler-Fusion system (Akoya Biosciences), according to the manufacturer’s instructions before H&E staining.

### Data processing

The default nuclei expansion-based segmentation from Xenium was found to be suboptimal with respect to the cell area and purity of cell types, in terms of marker genes, an observation similar to that of ref. ^51^. To find the optimal cell segmentation for the Xenium data, the default segmentation was refined using Baysor (v.0.6.2)^19^ with confidence in prior probability set to 0.5. To exclude artifacts, cells from the tissue regions that were excessively blurred or showed potentially noisy regions (such as folded and overlapping tissues) were removed. In addition the cells belonging to spots outside of the biopsy were excluded. Finally, cells that expressed fewer than five unique genes were removed.

Published single-cell reference data from the kidney^13^ were used to train a machine learning classifier. In the reference data 472 genes were common with the gene panel. The reference data and processed Xenium data were subsetted to include only the genes that were common between them both. The resulting datasets were log(normalized) using scanpy.pp.normalize_total and scanpy.pp.log1p methods from Scanpy (v.1.10.1). The target_sum was set to 1,000. LogisticRegressionCV class from scikit-learn (v.1.3.1) was used to train for the classification task, with the input being the normalized count matrix of the reference data and the target being the cell-type labels. The trained model was applied to the similarly normalized Xenium data and the labels were transferred to the original processed object.

### Defining the glomerular domain

To identify the glomerular niches based on the spatial transcriptome data, we used NichePCA^20^. Briefly, NichePCA constructs a spatial graph based on the spatial coordinates of cells and then calculates the mean aggregation of gene expressions for adjacent cells. Next, PCA is performed to reduce the dimensionality of the aggregated expressions and finally Leiden clustering is used to identify the spatial domains; here it resulted in the definition of glomeruli (Fig. 2a). Furthermore, cells showing a fibrotic signature inside the glomeruli regions were labeled fibrotic mesangial cells.

### Cell–cell interaction analysis

We used CellChat (v.2.1.2)^23^ to analyze cell–cell interactions, which utilizes the *x* and *y* coordinates of the cells available from spatial transcriptomic datasets to infer cell–cell communication, taking into account the spatial proximity of cells to infer probability of communication between them. As the 63 biopsy samples in this analysis were distributed across 8 slides, we added offset values to the *x* coordinates of cells to all slides except the first, thereby generating unique *x* and *y* coordinates for each slide to ensure that spatial interactions were inferred only among cells on the same slide.

Next, we generated separate CellChat objects for each of the four categories of samples (control, SLE, ANCA and anti-GBM) using the createCellChat function. We then set the ligand–receptor interaction database to include only ‘Secreted Signaling’, using the subsetDB function. Next, we preprocessed the expression data for cell–cell communication analysis using the identifyOverExpressedGenes and identifyOverExpressedInteractions functions. To compute the communication probability and infer cellular communication network, we used the computeCommunProb function with the truncatedMean method, applying a trim value of 0.1 to compute the average gene expression and set the parameters interaction.range to 250 and contact.range to 10. The cell–cell communication at a signaling pathway level was inferred using the computeCommunProbPathway function and aggregated cell–cell communication network combined using the aggregateNet function. Next, we calculated the difference in the interaction weight matrices for the cGN conditions over control, to visualize the difference seen in the cGN conditions with respect to the communication network that exists in control (Fig. 4a). The change in interaction weights for each of the three diseases individually over control is shown in Extended Data Fig. 6a.

To investigate all incoming signaling directed toward PECs, we used the subsetCommunication function to obtain a data frame of significant cell–cell interactions from each CellChat object. This data frame contains detailed information on significant inferred cell–cell communications. We then combined the data from the control and disease conditions into a comprehensive data frame representing all interactions. This data frame was then used for visualizing all incoming signaling directed toward PECs, which primarily included PDGF and TGFβ pathway interactions (Fig. 4b). The signals for each of the disease types individually is shown in Extended Data Fig. 6b.

### Data integration, integrating transcript and protein data

The transcriptome data were used as the fixed reference, whereas the proteomics and H&E staining data were transformed to align with them. In particular, for the transcriptomics and proteomics data, DAPI staining was used and the other protein channels were transformed accordingly. For image registration, we cropped the. tiff image files around each sample and applied the nonrigid registration workflow available in VALIS v.1.1.0^27^. The quality of registration was assessed by visualizing stacked images in FIJI v.1.54 (10.5281/zenodo.5256255). After registration, protein expression levels were assigned to individual cells. This was done by summing the pixel intensities of proteins located within the cell boundaries (using a shapely Python package), as defined by the cell segmentation algorithm.

### Clustering

We performed unsupervised clustering of ROIs using *K*-means clustering. The optimal number of clusters was determined using the elbow method implemented in the Yellowbrick package (v.1.5)^52^, which identified *K* = 5 as optimal, resulting in clusters containing 75, 119, 158, 210 and 220 ROIs. As two clusters were predominantly composed of control samples, these were merged to create a final set of four clusters labeled C1–C4, containing 194, 220, 210 and 158 ROIs, respectively.

### Pseudotime diffusion analysis

The raw counts of each ROI (unique glomerular + periglomerular areas) were summed to create pseudo-bulk ROIs. The resulting counts were normalized to sum to 10,000 and log(normalized) using preprocessing.normalize_total and preprocessing.log1p methods from Scanpy (v.1.10.1)^53^. Pseudotime computation was performed using the diffusion pseudotime method^54^ with a starting point as a random control ROI. Pseudotime was further divided into four clusters termed ‘quadrants’ using Jenkins’ natural breaks optimization with Python package jenkspy (v.0.4.1)^55^.

### Correlation analysis

ROIs were grouped according to their corresponding biopsy or patient identifiers. For each biopsy, the median PC1 value across all ROIs associated with that sample was calculated. This resulted in a single representative PC1 value per patient biopsy. We then tested the association between the median PC1 value and relevant clinical parameters using Spearman’s correlation. Specifically, we tested correlations between median PC1 and eGFR, albuminuria and age for all patients. In addition, correlation between median PC1 and ANCA renal risk score was calculated for patients with ANCA-associated vasculitis. Spearman’s correlation coefficient was computed using scipy.stats.spearmanr of the Python package SciPy.

### Drug prediction

Drug–target interaction data were extracted from the DrugBank database and processed to create a mapping of therapeutic compounds to their molecular targets and mechanisms of action. Pathway gene sets were defined using curated collections from MSigDB: REACTOME_SIGNALING_BY_PDGFR_IN_DISEASE for PDGF signaling (supplemented with PDGF ligands and receptors) and HALLMARK_TGF_BETA_SIGNALING for TGFβ signaling (which already included TGFβ ligands and receptors). To prioritize drugs, we performed drug scoring taking into account drug targets as well as drug actions. The base score quantified the pathway coverage of each drug *d* as ${S}_{{\rm{b}}}=|{T}_{d}\cap {G}_{p}|/|{G}_{p}|$, where *T*_*d*_ represents the set of targets of drug *d* and *G*_*p*_ represents the pathway gene set. Drug action relevance was quantified as ${S}_{{\rm{a}}}={\sum }_{g\in {T}_{d}\cap {G}_{p}}w({a}_{d,g})$, where $w({a}_{d,g})$ represents the action weight of a drug *d* on gene *g*, ${w(a}_{d,g})=1$ for potential inhibitors or antagonists and ${w(a}_{d,g})=0.5$ for potential binders, activators or agonists. The final prediction score combined target coverage with action-weighted relevance as ${S}_{\rm{f}}={S}_{\rm{b}}\times \left(1+{S}_{\rm{a}}/{\max} (1,\vert{T}_{d}\cap {G}_{p}\vert)\right.$. Drugs were filtered using a minimum score threshold of 0.1 and ranked by final prediction scores.

### SnRNA-seq

For snRNA-seq, snap-frozen biopsies were thawed and chopped using a fresh razor blade. After adding 200 µl of lysis buffer (1 mM dithiothreitol, 0.1% Triton X-100, 0.2 U µl^−1^ of SUPERase-In RNase Inhibitor and 0.4 U µl^−1^ of RNasin Plus Ribonuclease Inhibitor in Nuclei PURE lysis buffer), the tissue was homogenized 60× using a pestle on ice. Next, the lysate was supplemented with an additional 3.8 ml of lysis buffer and incubated on ice for 25 min. Lysis efficiency was assessed using an optical microscope. After incubation, a 30-µm strainer was used to filter the lysate and washed with 1 ml of lysis buffer. The lysate was centrifuged at 500*g* (5 min at 4 °C) and the supernatant was discarded. Then, 5 ml of washing buffer (0.01% bovine serum albumin (BSA), 0.4 U µl^−1^ of SUPERase-In RNase Inhibitor and 0.4 U µl^−1^ of RNasin Plus Ribonuclease Inhibitor in PBS) was added to the pellet and transferred to a Falcon tube coated with 0.1% BSA in PBS and centrifuged (500*g* for 5 min at 4 °C). Resuspension buffer (1% BSA and 0.4 U µl^−1^ of RNasin Plus Ribonuclease Inhibitor in PBS) was added to the pellet. Nuclei, 40,000, were loaded on to a Chip G for GEM generation using the Chromium Single Cell 3ʹ v.3.1 kit (10x Genomics). Reverse transcription, barcoding, complementary DNA amplification and purification for library preparation were performed as specified in the manufacturer’s protocol. Sequencing was performed on a NovaSeq 6000 platform (Illumina) at a target read depth of 25,000 by Novogene.

Cell Ranger software (v.7.1.0, 10x Genomics) was used to process cellular barcodes and align reads to the GRCh38-2020-A reference genome. QC and preprocessing were performed using Scanpy (v.1.10.1) and Python (v.3.11.6). Nuclei expressing <50 genes were excluded. In addition, low-quality cells with >10% mitochondrial genes were filtered out. The raw count data were normalized to 10,000 reads per nucleus, log(transformed) (log(1*P*)), batch corrected and integrated with Scanpy using the 2,000 most highly variable genes. Clustering was performed using the Leiden algorithm with resolution 0.8 and cell types were annotated using a logistic regression classifier. Clusters with a median annotation confidence score <0.6 were removed from downstream analysis.

### Mouse experiments

Experimental cGN was induced by injection i.p. of nephrotoxic sheep serum in 8–12-week-old male and female C57B6/J mice randomly assigned to the respective group^37^. Mice were kept under specific pathogen-free conditions with a constant dark-to-light cycle, temperature 20–24 °C and humidity 40–65%. After cGN induction, mice were treated with either nintedanib 0.9 mg per mouse p.o. daily^56^ from day 5 to day 9 or fostamatinib 0.8 mg per mouse i.p. twice daily^57^ from day 10 to day 19, as indicated. Mice with no histological features of kidney disease were excluded from the analysis. All animal protocols were approved by the local ethics committee (Behörde für Justiz und Verbraucherschutz Hamburg) and in accordance with national guidelines. No statistical methods were used to predetermine sample sizes but our sample sizes are similar to those reported in previous publications^37^.

### Immunofluorescence

#### Human biopsies

Paraffin sections were deparaffinized and antigen retrieval was performed for pERK staining (DAKO, pH 6, cat. no. S2369) and pSMAD3 staining (DAKO, pH 6, cat. no. S1699) by steamer boiling for 40 min. Unspecific binding was blocked in 5% horse serum (Vector) and 0.05% Triton X-100 (Sigma-Aldrich) for 30 min. Primary antibodies used for immunofluorescence microscopy of human kidney were as follows: for staining PDGF pathway activity in PECs: rabbit pERK1/2 (1:50, Cell Signaling, cat. no. 4344S), rat CD44 (1:100, BD Pharmingen, cat. no. 550538), rabbit claudin-1 (1:100, Invitrogen, cat. no. 717800) and guinea-pig synaptopodin (1:200, Synaptic Systems, cat. no. 163004) were combined. For visualization of TGFβ pathway activity in PECs, a combination of rabbit anti-SMAD3 pS423 or pS425 (1:100, Abcam, cat. no. ab11882), ms-Pax8 (1:50, Cell Signaling, cat. no. 28556S), guinea-pig nephrin (1:200, Progene, cat. no. GP-N2) and guinea-pig synaptopodin (1:200) was applied in blocking buffer. Primary antibody incubations (overnight, 4 °C) were followed by incubation for 30 min at room temperature in blocking buffer with the secondary antibodies coupled with AF488, Cy3, AF594, Cy5 and AF790 (all at 1:200, affinity-purified donkey antibodies by Jackson ImmunoResearch). Nuclei were visualized using Hoechst (1:1,000, Invitrogen Hoechst 33342, cat. no. H3570).

#### Mouse kidneys

Paraformaldehyde (4%)-fixed kidney cortex was embedded in paraffin for high-resolution confocal microscopy. The 3-µm paraffin sections were cut on a rotation microtome, deparaffinized and underwent antigen retrieval for pERK staining, and proliferating cell nuclear antigen (PCNA; DAKO, pH 9, cat. no. S2367), and pSMAD3 staining was performed by steamer boiling for 40 min. For the SMA staining the 3-µm paraffin sections were digested by proteinase K (Sigma-Aldrich, cat. no. vP6556, 1:2,000 in PBS) for 15 min at 37 °C. The primary antibodies used for immunofluorescence microscopy of mouse kidneys are as follows: for staining of glomerular fibrosis, rabbit SMA (1:400, Abcam, cat. no. ab5694) and guinea-pig synaptopodin (1:200) were combined. For staining of PEC proliferation, mouse PCNA (1:50, CalBioChem, cat. no. NA03), rabbit claudin-1 (1:100), goat Pax8 (1:50, Abcam, cat. no. ab13611) and guinea-pig synaptopodin (1:200) were combined. For staining of PDGF pathway activity in PECs, rabbit pERK1/2 (1:50, Cell Signaling, cat. no. 4344S), rat CD44 (1:100, BD Pharmingen, cat. no. 550538), goat Pax8 (1:50), rabbit claudin-1 (1:100) and guinea-pig synaptopodin (1:200) were combined. For staining of TGFβ pathway activity in PECs, a combination of rabbit SMAD3 pS423 or pS425 (1:100), goat Pax8 (1:50) and guinea-pig synaptopodin (1:200) was used. Primary antibody incubations (overnight, 4 °C) were followed by incubation for 30 min at room temperature in blocking buffer with secondary antibodies coupled to AF488, Cy3, AF594, Cy5 and AF790 (all at 1:200). Nuclei were visualized using Hoechst (1:1,000).

Staining was evaluated with an LSM980 equipped with an Airyscan 2 and NIR Laser for high-resolution confocal microscopy using ZENblue software (all Zeiss).

### Statistics and reproducibility

All measurements were taken from distinct samples. Data distribution was assumed to be normal but was not formally tested. Evaluation of crescents and glomerulosclerosis was performed in a blinded fashion. Differential gene expression analysis of PC clusters: Xenium slide IDs were treated as a covariate in the differential gene expression analysis between PC clusters and pseudotime quadrants. Disease was not included because not all clusters had all diseases represented. Sample size per cluster did not differ much and this covariate did not need to be included.

### Reporting summary

Further information on research design is available in the Nature Portfolio Reporting Summary linked to this article.

## Online content

Any methods, additional references, Nature Portfolio reporting summaries, source data, extended data, supplementary information, acknowledgements, peer review information; details of author contributions and competing interests; and statements of data and code availability are available at 10.1038/s41590-025-02291-8.

## Supplementary information

Reporting Summary

Supplementary Table 1 Gene panel designed for the identification of different kidney cells, immune cells and glomerulonephritis-driving genes.

## Data Availability

Gene expression data generated in this study are deposited in the National Center for Biotechnology Information Gene Expression Omnibus database under accession nos. GSE294965 for spatial analysis and GSE303481 for snRNA-seq. External datasets containing scRNA-seq and snRNA-seq data from normal, acute kidney injury and chronic kidney disease kidney samples^13^ are publicly accessible through the CellXGene data portal (https://cellxgene.cziscience.com/collections/bcb61471-2a44-4d00-a0af-ff085512674c) and the Kidney Precision Medicine Project repository (https://atlas.kpmp.org/repository/).

## Code availability

The code to process and analyze the data is available via GitHub at https://github.com/imsb-uke/xenium-cgn.
