# Supplementary material for: Spatiotemporal interaction of immune and renal cells controls glomerular crescent formation in autoimmune kidney disease
Source: Nat Immunol. 2025 Sep 30;26(11):1977–88. doi: 10.1038/s41590-025-02291-8 (PMC12571875; doi:10.1038/s41590-025-02291-8)
Supplement: Supplementary file 1 — Reporting Summary [file 41590_2025_2291_MOESM1_ESM.pdf]

# nature portfolio

Corresponding author(s): Ulf Panzer, Stefan Bonn, Christian F. Krebs

Last updated by author(s): Jul 30, 2025

# Reporting Summary

Nature Portfolio wishes to improve the reproducibility of the work that we publish. This form provides structure for consistency and transparency in reporting. For further information on Nature Portfolio policies, see our Editorial Policies and the Editorial Policy Checklist.

## Statistics

For all statistical analyses, confirm that the following items are present in the figure legend, table legend, main text, or Methods section.

| n/a | Confirmed | |
|---|---|---|
| ☐ | ☒ | The exact sample size (*n*) for each experimental group/condition, given as a discrete number and unit of measurement |
| ☐ | ☒ | A statement on whether measurements were taken from distinct samples or whether the same sample was measured repeatedly |
| ☐ | ☒ | The statistical test(s) used AND whether they are one- or two-sided<br>*Only common tests should be described solely by name; describe more complex techniques in the Methods section.* |
| ☐ | ☒ | A description of all covariates tested |
| ☒ | ☐ | A description of any assumptions or corrections, such as tests of normality and adjustment for multiple comparisons |
| ☒ | ☐ | A full description of the statistical parameters including central tendency (e.g. means) or other basic estimates (e.g. regression coefficient) AND variation (e.g. standard deviation) or associated estimates of uncertainty (e.g. confidence intervals) |
| ☒ | ☐ | For null hypothesis testing, the test statistic (e.g. *F*, *t*, *r*) with confidence intervals, effect sizes, degrees of freedom and *P* value noted<br>*Give P values as exact values whenever suitable.* |
| ☒ | ☐ | For Bayesian analysis, information on the choice of priors and Markov chain Monte Carlo settings |
| ☒ | ☐ | For hierarchical and complex designs, identification of the appropriate level for tests and full reporting of outcomes |
| ☐ | ☒ | Estimates of effect sizes (e.g. Cohen's *d*, Pearson's *r*), indicating how they were calculated |

*Our web collection on statistics for biologists contains articles on many of the points above.*

## Software and code

Policy information about availability of computer code

| | |
|---|---|
| Data collection | Whole slide imaging: Leica Application Suite X software (v3.7.4.23463), Xenium Onboard Analysis v1.7 (10x Genomics), Phenocycler Fusion 2.0 software 1.0.8 (Akoya), ZENblue software (Zeiss) |
| Data analysis | Cell segmentation refinement: Baysor (v0.6.2), Xenium data normalization and processing: Scanpy (v1.10.1), cell type classification: scikit-learn (v1.3.1), nichePCA (v0.0.1), cell-cell interaction analysis: CellChat (v2.1.0), image registration: Fiji (v1.54), Napari (v0.4.19.post1) and VALIS (v1.1.0), geometric operations: Shapely (2.0.5), genome alignment of single-nuc data: Cell Ranger (v7.1.0, 10x Genomics), single-nuc data normalization and processing: Seurat (v4.9.9). Correlation analysis: SciPy (v1.11.3). All R and Python packages were run using R (v4.3.1) and Python (3.11.1). hGithub repository: ttps://github.com/imsb-uke/xenium-cgn. |

For manuscripts utilizing custom algorithms or software that are central to the research but not yet described in published literature, software must be made available to editors and reviewers. We strongly encourage code deposition in a community repository (e.g. GitHub). See the Nature Portfolio guidelines for submitting code & software for further information.

# Data

Policy information about availability of data

All manuscripts must include a data availability statement. This statement should provide the following information, where applicable:

- Accession codes, unique identifiers, or web links for publicly available datasets
- A description of any restrictions on data availability
- For clinical datasets or third party data, please ensure that the statement adheres to our policy

All gene expression data generated in this study is provided via the National Center for Biotechnology Information Gene Expression Omnibus database (GSE294965 for spatial analysis, GSE303481 for snRAN-seq). External datasets containing single-cell and single-nucleus RNA-sequencing data from normal, acute kidney injury (AKI), and chronic kidney disease (CKD) kidney samples13 are publicly accessible through the CellXGene data portal (https://cellxgene.cziscience.com/collections/bcb61471-2a44-4d00-a0af-ff085512674c) and the Kidney Precision Medicine Project (KPMP) repository (https://atlas.kpmp.org/repository/).

# Research involving human participants, their data, or biological material

Policy information about studies with human participants or human data. See also policy information about sex, gender (identity/presentation), and sexual orientation and race, ethnicity and racism.

| | |
|---|---|
| Reporting on sex and gender | Due to the sample size, sex- and gender-based analyses were not performed. Information about the sex of the the patients is provided in Table 1. Self reported sex and biological sex were identical in the exploratory and treatment cohort. |
| Reporting on race, ethnicity, or other socially relevant groupings | Data on race, ethnicity or other socially relevant groupings were not included. |
| Population characteristics | This information is provided in Table 1. |
| Recruitment | All samles analyzed  in this study were included in the Hamburg Glomerulonephritis Registry. Informed consent was obtained from all patients. |
| Ethics oversight | This study was approved by the Institutional Reviewing Board (IRB) of the University Medical Center Hamburg-Eppendorf and the Ethik-Kommission der Ärztekammer Hamburg (local ethics committee of the chamber of physicians in Hamburg) |

Note that full information on the approval of the study protocol must also be provided in the manuscript.

# Field-specific reporting

Please select the one below that is the best fit for your research. If you are not sure, read the appropriate sections before making your selection.

☒ Life sciences ☐ Behavioural & social sciences ☐ Ecological, evolutionary & environmental sciences

For a reference copy of the document with all sections, see nature.com/documents/nr-reporting-summary-flat.pdf

# Life sciences study design

All studies must disclose on these points even when the disclosure is negative.

| | |
|---|---|
| Sample size | 57 samples from patients with RPGN, 6 control samples. |
| Data exclusions | No data was excluded. |
| Replication | One repetition of each animal experiment was performed. |
| Randomization | No randomization was performed. |
| Blinding | Histologies were scored in a blinded fashion. |

# Reporting for specific materials, systems and methods

We require information from authors about some types of materials, experimental systems and methods used in many studies. Here, indicate whether each material, system or method listed is relevant to your study. If you are not sure if a list item applies to your research, read the appropriate section before selecting a response.

## Materials & experimental systems

| n/a | Involved in the study | |
|---|---|---|
| ☐ | ☒ | Antibodies |
| ☒ | ☐ | Eukaryotic cell lines |
| ☒ | ☐ | Palaeontology and archaeology |
| ☐ | ☒ | Animals and other organisms |
| ☐ | ☒ | Clinical data |
| ☒ | ☐ | Dual use research of concern |
| ☒ | ☐ | Plants |

## Methods

| n/a | Involved in the study | |
|---|---|---|
| ☒ | ☐ | ChIP-seq |
| ☒ | ☐ | Flow cytometry |
| ☒ | ☐ | MRI-based neuroimaging |

# Antibodies

**Antibodies used**

Anti-Hu Beta-actin(AKYP0072)-BX117—Alexa Fluor™ 488 for PhenoCycler
Anti-Hu/Mu Ki67(AKYP0052)-BX047—Atto 550 for PhenoCycler
(Antibodies for PhenoCycler were used according to the manufacturer´s insturctions.)
Anti-CD44 (1:100, polyclonal, BD Pharmingen #550538)
Anti-Claudin1 (1:100, polyclonal, Invitrogen #717800)
Anti-Synaptopodin (1:200, polyclonal, Synaptic Systems #163004)
Anti-SMAD3 pS423/pS425 (0.5 mg/mL, clone EPR18021, Abcam #ab11882)
Anti-Pax8 (1:50, clone BC12, Cell Signaling #28556S)
Anti-Nephrin (1:200, polyclonal, Progene, #GP-N2)
Anti-SMA (1:400, polyclonal, Abcam #ab5694)
Anti-PCNA (1:50, clone NM-11, CalBioChem #NA03)
Anti-Pax8 (1:50, polyclonal, Abcam #ab13611)
akoyabio.com; bdbiosciences.com; abcam.com; thermofisher.com; sysy.com; progen.com; cellsignal.com; sigmalaldrich.com

**Validation**

n/a

# Animals and other research organisms

Policy information about studies involving animals; ARRIVE guidelines recommended for reporting animal research, and Sex and Gender in Research

**Laboratory animals**

C57BL/6 mice. Mice were maintained in specific pathogen-free conditions with controlled humidity and temperature with a light/ dark cycle of 12h each.

**Wild animals**

No wild animals were used.

**Reporting on sex**

Male and female animals were used. The experiments were not powered to perform sex-based analyses.

**Field-collected samples**

No field-collected samples were used in this study.

**Ethics oversight**

Experiments with mice followed the national guidelines, and local ethics committees (Behörde für Justiz und Verbraucherschutz Hamburg) approved the research protocols.

Note that full information on the approval of the study protocol must also be provided in the manuscript.

# Clinical data

Policy information about clinical studies

All manuscripts should comply with the ICMJE guidelines for publication of clinical research and a completed CONSORT checklist must be included with all submissions.

**Clinical trial registration**

n/a

**Study protocol**

n/a

**Data collection**

Clinical data was obtained from the Hamburg Glomerulonephritis Registry.

**Outcomes**

n/a

## Plants

| | |
|---|---|
| Seed stocks | *Report on the source of all seed stocks or other plant material used. If applicable, state the seed stock centre and catalogue number. If plant specimens were collected from the field, describe the collection location, date and sampling procedures.* |
| Novel plant genotypes | *Describe the methods by which all novel plant genotypes were produced. This includes those generated by transgenic approaches, gene editing, chemical/radiation-based mutagenesis and hybridization. For transgenic lines, describe the transformation method, the number of independent lines analyzed and the generation upon which experiments were performed. For gene-edited lines, describe the editor used, the endogenous sequence targeted for editing, the targeting guide RNA sequence (if applicable) and how the editor was applied.* |
| Authentication | *Describe any authentication procedures for each seed stock used or novel genotype generated. Describe any experiments used to assess the effect of a mutation and, where applicable, how potential secondary effects (e.g. second site T-DNA insertions, mosiacism, off-target gene editing) were examined.* |
